# Effects of Daytime Electric Light Exposure on Human Alertness and Higher Cognitive Functions: A Systematic Review

**DOI:** 10.3389/fpsyg.2021.765750

**Published:** 2022-01-05

**Authors:** Mushfiqul Anwar Siraji, Vineetha Kalavally, Alexandre Schaefer, Shamsul Haque

**Affiliations:** ^1^Department of Psychology, Jeffrey Cheah School of Medicine and Health Sciences, Monash University Malaysia, Subang Jaya, Malaysia; ^2^Department of Electrical and Computer Systems Engineering, School of Engineering, Monash University Malaysia, Subang Jaya, Malaysia; ^3^School of Medical and Life Sciences, Sunway University, Subang Jaya, Malaysia

**Keywords:** short-wavelength dominant light, higher intensity white light, subjective alertness, reaction time, higher cognitive functions

## Abstract

This paper reports the results of a systematic review conducted on articles examining the effects of daytime electric light exposure on alertness and higher cognitive functions. For this, we selected 59 quantitative research articles from 11 online databases. The review protocol was registered with PROSPERO (CRD42020157603). The results showed that both short-wavelength dominant light exposure and higher intensity white light exposure induced alertness. However, those influences depended on factors like the participants’ homeostatic sleep drive and the time of day the participants received the light exposure. The relationship between light exposure and higher cognitive functions was not as straightforward as the alerting effect. The optimal light property for higher cognitive functions was reported dependent on other factors, such as task complexity and properties of control light. Among the studies with short-wavelength dominant light exposure, ten studies (morning: 3; afternoon: 7) reported beneficial effects on simple task performances (reaction time), and four studies (morning: 3; afternoon: 1) on complex task performances. Four studies with higher intensity white light exposure (morning: 3; afternoon: 1) reported beneficial effects on simple task performance and nine studies (morning: 5; afternoon: 4) on complex task performance. Short-wavelength dominant light exposure with higher light intensity induced a beneficial effect on alertness and simple task performances. However, those effects did not hold for complex task performances. The results indicate the need for further studies to understand the influence of short-wavelength dominant light exposure with higher illuminance on alertness and higher cognitive functions.

## Introduction

Besides its role in vision, light also influences several physiological and psychological processes – known as non-image-forming (NIF) effects of light. The “Commission Internationale de l’Eclairage” (CIE) later adopted the term of “intrinsically photosensitive retinal ganglion cell (ipRGCs) influenced light (IIL) responses” (CIE, 2018) to characterize these effects. “Illuminating Engineering Society” (IES) categorized IIL responses as circadian, neuroendocrine and neurobehavioral responses (IES, 2018). Circadian responses entrain our internal 24-h clock to earth’s 24-h rotation (de Kort, 2019). Neuroendocrine responses control how the brain regulates hormone expression, especially melatonin secretion (Lockley, 2008; Cajochen and Chellappa, 2016). Neurobehavioral responses indicate the relationship between the nervous system and human behavior. They typically include psychological processes such as alertness (Lockley et al., 2006; Cajochen, 2007), mood (Xiao et al., 2021), cognitive performance (Alkozei et al., 2017) as well as different physiological states like heart rate (Cajochen et al., 2005; Askaripoor et al., 2018) and body temperature (te Kulve et al., 2016).

The melanopsin enriched intrinsically photoreceptive retinal ganglion cells (ipRGCs) play a significant role in IIL responses (Berson et al., 2002). The ipRGCs have a non-visual retinal-hypothalamic connection with the mammalian circadian oscillator “Suprachiasmatic Nuclei” (SCN), thus regulating the IIL responses (Gooley et al., 2003). CIE (2018) has characterized the spectral sensitivity curve peaking at 490 nm for ipRGCs. At least three subtypes of ipRGCs are reported in humans with distinct responses to light (Mure et al., 2019). These three subtypes of ipRGCs have different sensitivities and responses to light and they are related to distinct target areas in the brain. The role played by other photoreceptors’ (S-, M-, L- cones and rods) in the IIL responses is still not fully understood (Gooley et al., 2010, 2012; Lucas et al., 2012). The ipRGCs can combine their own intrinsic inputs, which are melanopsin-driven in nature, with extrinsic inputs originated from rods and cones (Smith et al., 2005). Thus, we can say ipRGCs, rods and cons may all influence visual and IIL responses in Houser et al. (2020) and Lucas et al. (2014).

Understanding the interaction of different light properties and their relationship with IIL responses is fundamental to maintain and facilitate the desired neurobehavioral, circadian, and neuroendocrine responses. However, from the current state of literature, it is not easy to find a one size fits all solution as there are many variables at play, including individual differences (chronotypes, sleep habit, homeostatic sleep drive), light’s spectral power distribution, temporal pattern (duration of light, time, and prior history) and spatial pattern (luminance distribution in the space) as well as the complexity of the task used in measuring IIL responses.

To contribute to a better understanding of IIL responses, we present in this paper a systematic review of daytime effects of electric light on our alertness and higher cognitive functions reported in studies published from 1806 to 2021. Before presenting our systematic review and its findings, we include hereafter an excursus on light metrology and IIL response circuitry.

### Light Metrology

**FIGURE 1:**
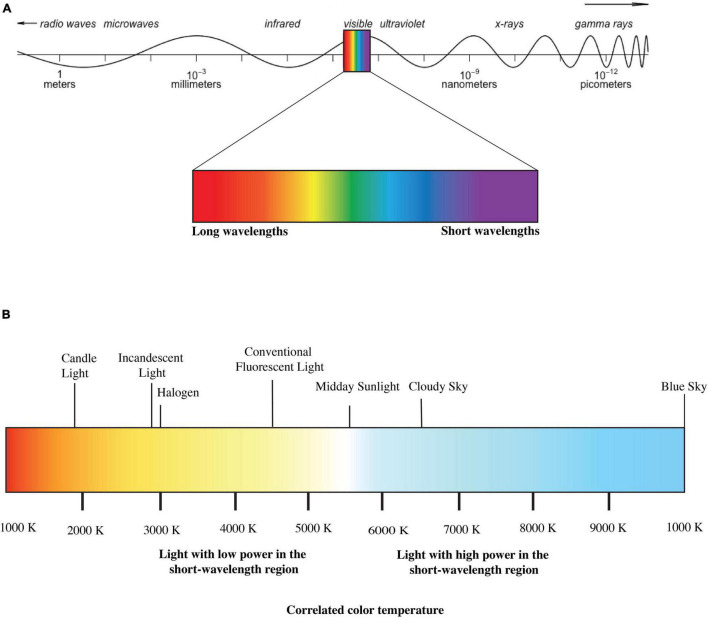
**(A)** Electromagnetic Spectrum and **(B)** correlated color temperature (CCT) of lights (disclaimer: figure is for illustration purpose only).

Light is electromagnetic radiation that can directly stimulate retinal photoreceptors of the visual system to form a visual sensation (CIE, 2019). Although this definition limits light only to visual responses, it is a widespread practice in IIL literature to use “light” to describe a particular proportion of optical radiation that might induce both visual and IIL responses (Houser et al., 2020). In this paper, we have used “light” following the latter convention. Figure 1A depicts a simplified illustration of electromagnetic spectrum. Conventionally different photometric and radiometric indices are used to describe different properties of light. Radiometry characterizes electromagnetic waves’ physical properties based on a scalar field where different quantities represent radiation direction (Rossi, 2019). Photometric quantities explicitly deal with only the visible portion of the energy spectrum (380–780 nm) and describe this visible proportion according to human photoreceptors’ sensitivity. The indices used in photometry are basically cone-dominated metrics. Correlated color temperature (CCT) is such a cone-dominated metric that defines the visible color characteristics of a light source according to a black body locus. The black body locus is the path line of the color of an incandescent black body in relation to the temperature change defined by the CIE-1931 chromaticity diagram (CIE, 1931). The more power it provides to the shorter-wavelength region of visible light, the higher the CCT (Figure 1B). However, these cone dominated matrices are not sufficient to quantify light properties for IIL responses as all photoreceptors (rods, cones and ipRGCs) may contribute to IIL responses and their contributions may vary depending on light’s spectrum, intensity, temporal pattern, spatial pattern and as well as individual differences (Lucas et al., 2014). To address this issue, CIE (2018) has published an international standard (S026:E:2018) with SI units such as α- opic equivalent daylight (D65) illuminances (α-opic EDIs) to account for spectral sensitivity functions more precisely in terms of the light exposures’ ability to stimulate each of the five α-opic retinal photoreceptors. CIE (2018) has also introduced a new “M/P ratio”- melanopic daylight (D65) efficacy ratio (MDER), which is the ratio of a test light’s melanopic equivalent daylight (D65) illuminance to its photopic illuminance (Schlangen and Price, 2021). A high MDER ratio indicates the light source’s higher capability to stimulate the ipRGCs.

### The Intrinsically Photosensitive Retinal Ganglion Cell-Influenced Light Responses

Neuroimaging studies showed that ipRGCs project intrinsic and/or extrinsic light information to different brain regions, including the master pacemaker-suprachiasmatic nucleus (SCN) via the retinohypothalamic tract (Gooley et al., 2003). Additionally, various hypothalamic regions also receive signals from ipRGCs, including ventral lateral preoptic area (VLPO), locus coeruleus (LC), dorsal pathway (DR) and lateral hypothalamic area (LH) (Vandewalle et al., 2009; Lok et al., 2018a). The ipRGCs also target the amygdala (AM), the superior colliculus (SC), periaqueductal gray (PAG) and the supraventricular zone (SPZ) (Gooley et al., 2003; Vandewalle et al., 2009).

**FIGURE 2:**
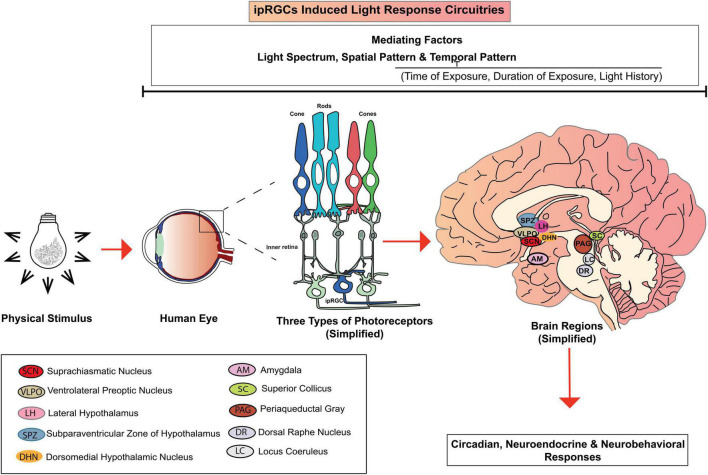
IpRGCs induced light response circuitries.

Lok et al. (2018a), in their review elaborately discussed the possible IIL response circuitry (Figure 2), especially for alerting responses. Our state of alertness is mostly controlled by the brain region associated with the thalamus (Aston-Jones, 2005) and is also subject to SCN regulation (Lok et al., 2018a). By light stimulation, ipRGCs can influence SCN controlled activity which in turn may influence our state of alertness. The nocturnal alerting effects are mostly contributed to the light’s ability to suppress melatonin through inputs from ipRGCs (Cajochen et al., 2005; Lockley et al., 2006; Houser et al., 2020). However, in the case of daytime light exposure, when the melatonin level is undetectable, alerting effects follow a different mechanism. As shown in Figure 2, different hypothalamic brain regions, including VLPO, LC, DR receive intrinsic and extrinsic inputs from the ipRGCs via direct projection from ipRGCs and/or indirect projection from SCN (Gooley et al., 2003; Moore et al., 2012). VLPO and LC are known to regulate our sleep regulation and arousal (Fort et al., 2009). When VLPO is inactive, alertness is promoted (Moore et al., 2012). Activation of LC promotes arousal, which in turn promotes an increased state of alertness (Berridge and Foote, 1991). Both LC and DR play a substantial role in wakefulness (Lee et al., 2005). In addition to the activation of different brain regions, daytime light exposure may also induce alertness by reducing homeostatic sleep drive (Rahman et al., 2014), which regulates our sleep cycle internally. As our waking hour increases, our homeostatic sleep drive increases. Daytime light exposure can reduce this sleep drive and makes us more alert.

### Lighting Variables

The IIL responses are highly dependent on the spectrum, spatial and temporal patterns (Houser et al., 2020), of which we will only be focusing on the spectrum and temporal patterns. Temporal pattern of light includes exposure duration, timing and prior history of light exposure. Studies included in our review have employed electric lights with different spectral properties: monochromatic short-wavelength light (Lockley et al., 2006; Rahman et al., 2014), polychromatic white light with narrow pass filter (Alkozei et al., 2017; Daneault et al., 2018), polychromatic white light with high power over the short-wavelength region (Askaripoor et al., 2018; te Kulve et al., 2018) or, higher intensity polychromatic white light to investigate their influence in IIL responses (Cajochen et al., 2000; Lok et al., 2018a). Table 1 summarizes the spectral properties of electric lights identified in this systematic review. Monochromatic light sources radiate only one wavelength, whereas polychromatic light radiates more than one peak wavelengths in the visible spectrum. When a polychromatic light radiates a mixture of more than one peak wavelength resulting in a white light, it is called polychromatic white light. Interference filters like narrow pass filters are used to suppress the long wavelengths of a polychromatic white light source. Polychromatic white light with high power in the short-wavelength region are basically lights with higher power over the blue region of the visible light spectrum. This systematic review clustered studies with monochromatic short-wavelength light, polychromatic white light with narrow pass filter, and polychromatic white light with high power in the short wavelength region under the label “short-wavelength dominant light exposure.” The group of studies that used polychromatic white light and investigated the differential influence of higher illuminance level on the IIL responses is clustered under the label “higher intensity white light exposure.”

**TABLE 1:** Different types of electric lights included in this systematic review.

Light description	Spectral properties
Monochromatic light	Light source that radiates only one wavelength in the visible spectrum.
Polychromatic light	Light source that radiates more than one peak wavelengths in the visible spectrum.
Polychromatic white light	Light source that radiates a mixture of more than one peak wavelengths in the visible spectrum resulting in white light.
Polychromatic white light with narrow pass filter	Polychromatic white light source with interference filters used to suppress the unwanted wavelengths.
Polychromatic white light with high power in the short-wavelength region	Polychromatic white light source with higher power in the blue wavelength region.

### Alertness and Higher Cognitive Functions

Alertness is the state of being awake, aware, attentive and prepared to act or react (Vandebos, 2007). Studies of attention defined alertness as achieving and maintaining a state of vigilance to respond adequately to a given stimulus (Oken et al., 2006; Shapiro et al., 2006). Alertness may also indicate a subjective experience of feeling alert (Schmidt et al., 2007). In IIL response literature, “alertness” is most commonly operationally defined by observable task performance such as faster reaction time in different cognitive tasks, or subjective evaluation of an individual’s current state of being awake. Cognitive tasks employed to measure alertness such as go-no-go and n-back tasks also incorporate different aspects of higher cognitive functions such as memory, executive functions, and inhibition. In addition, considering the IIL response circuitry that includes different brain regions related to physiological arousal, measures like electroencephalograph (EEG), electrooculography (EOG) and electrodermal activity (EDA) are also used to measure neurophysiological correlates of alertness (Cajochen, 2007).

Due to its varied nature, it is challenging to categorize the measures of alertness used in IIL response literature. Schmidt et al. (2007) classified the subjective reporting of feeling alert as subjective alertness and cognitive tasks that measure different cognitive domains, including alertness as objective performance measures. However, Lok et al. (2018a), in their work further differentiate among the objective performance measures as sustained attention tasks and executive performance tasks. Sustained attention tasks relied primarily on alertness, whereas executive performance tasks in addition to alertness may require higher-order cognitive functions (inhibition, executive functions) as well (Lok et al., 2018a). Cognitive tasks include in this review measure sustained attention, inhibition, executive functioning, and memory. In this systematic review, we considered self-reported measures of alertness as subjective measures of alertness. Neurophysiological correlates of alertness and sustained attention tasks are considered objective alertness measures. Performance in cognitive tasks that require higher-order cognitive functions is clustered under the label “higher cognitive functions.” Table 2 briefly summarizes different neurophysiological correlates and cognitive measures of alertness and higher cognitive functions. In EEG, both the power density of the alpha band (8–10 Hz) and theta band (10–13 Hz) activities of our brain were employed to measure attentional processing (Askaripoor et al., 2019). EOG used indices including slow eye movement (SEM) and eye blink to measure neurophysiological correlates of alertness (Phipps-Nelson et al., 2003; Lok et al., 2018b). ECG used heart rate index (Askaripoor et al., 2019) and EDA used skin conductance level (SCL) (Smolders and de Kort, 2017) to measure neurophysiological correlates of alertness. Sustained attention tasks measuring objective alertness used reaction time as an alertness index. And cognitive tasks measuring higher cognitive functions used reaction time and accuracy as performance indices.

**TABLE 2:** Different physiological and cognitive measures of alertness and higher cognitive functions.

Domain	Measure	Description	Index
**Subjective alertness**	Karolinska Sleepiness Scale	A 9-point scale to measure subjective sleepiness (Akerstedt and Gillberg, 1990).	Subjective reporting
**Objective alertness**			
Sustained attention task	Psychomotor vigilance test (PVT)	A computer based 5–10 min sustained attention task to measure alertness (Lim and Dinges, 2008). Can be of visual (Grant et al., 2021) or auditory mode (Burattini et al., 2019).	Reaction time
	Attention network test (ANT)	Measures alerting, orienting, and executive networks (Fan et al., 2002). The alerting network is reported under objective alertness.	Reaction time
Neurophysiological correlates of alertness	Electroencephalography (EEG)	Records electrical activity of human brain (Askaripoor et al., 2019).	Power density of alpha and/or theta band
	Electrocardiogram (ECG)	Records electrical activity of heart (Choi et al., 2019).	Heart rate
	Electrooculography (EOG)	Records eyelid and eye movement pattern (Lok et al., 2018b).	Slow eye movements (SEM) Eye blink
	Electrodermal activity (EDA)	Records electrical activity of human skin (Smolders and de Kort, 2017).	Skin conductance level (SCL)
**Higher cognitive functions**			**Indices**
	Addition task	Measures concentration, working memory and mathematical ability of an individual (Grant et al., 2021).	Accuracy
	N-back tasks	Measures working memory (Kirchner, 1958; Mackworth, 1959; Choi et al., 2019) where a list of stimuli is presented with an interval of several seconds. The task is to decide whether the present stimulus matches the one displayed n trials back (*n* = 1,2,3…*n*).	Reaction time Accuracy
	Purdue visualization rotation	A subtest of the Purdue spatial visualization test battery (Bodner and Guay, 1997) to assess the mental rotation ability.	Reaction time Accuracy
	Tloaddback	Combines 1-back task and parity digit decision task to assess an individual’s working memory (Borragán et al., 2016).	Accuracy
	Digit span	A verbal measure of working memory capacity. The three commonly used variants are forward digit span, back ward digit span and sequential digit span (Huiberts et al., 2015).	Accuracy
	Paced visual serial addition task (PVSAT)	A measure of attention and information processing speed where the task is to add the last digit to the previous digit presented (Gronwall, 1977).	Reaction time Accuracy
	Lexical decision task	A measure of long-term memory where participants decide whether a combination of letters are real word or not (Meyer and Schvaneveldt, 1971).	Accuracy
	Long term memory test	A measure of long term memory comprising one learning phase and one recall phase of a list of emotional words (Zhu et al., 2019).	Accuracy
	California verbal learning test II (CVLT-II)	An individually administered verbal memory test with immediate recall, short-delay and long-delay free recall components (Woods et al., 2006).	Accuracy
	Go-no-go task	Measures an individual’s processing speed and inhibitory capacity where participants are required to respond to a certain stimulus and make no response for the others (Smolders and de Kort, 2014).	Reaction time Accuracy
	Task switching	Measures a person’s ability to shift attention between tasks (Kaida et al., 2013).	Reaction time Accuracy
	Letter cancellation task	A measure of inhibitory capacity where the task is to cancel out a particular letter repeatedly (Smolders and de Kort, 2017).	Accuracy
	Attention network test	Measures alerting, orienting, and executive networks (Fan et al., 2002). In our systematic review we clustered the executive network of ANT under higher cognitive functions.	Reaction time Accuracy
	Flanker task	A measure of executive function to assess sustain attention and inhibition where a directional response is made to a central target stimulus (Eriksen and Eriksen, 1974).	Reaction time Accuracy
	Driving task	A simulator to test driving performance (Rodriguez-Morilla et al., 2018).	Reaction time Accuracy
	Continuous performance test	A standardized computer test to measure a person’s sustained and selective attention (Riccio et al., 2002).	Reaction time Accuracy
	Auditory odd-ball	Accompanied by p3000 event related potential, auditory odd-ball generally measures attention allocation (Polich and Kok, 1995). Here the presentation of a sequence of auditory stimuli is infrequently interrupted by another unique stimulus and the reaction of participants to this interruption is recorded.	Reaction time
	Multi-attribute task battery (MATB-II)	A computer-based task to assess long-term concentration performance which includes monitoring task, resource management task and tracking task (Ye et al., 2018).	Accuracy

In this systematic review, we considered the reaction time index in the latter group as easy task performance and accuracy as complex task performance. Lastly, for the attention network task (ANT), we only report the attention network as the measure of objective alertness. The executive control network of ANT is reported as “higher cognitive functions.”

### The Relevance of This Systematic Review

There are already several reviews focusing on the alerting effects of electric light (Figueiro et al., 2017; Lok et al., 2018a; Souman et al., 2018) and mood (Xiao et al., 2021) – none of these reviews except Lok et al. (2018a) incorporated higher cognitive functions. However, these authors focused their review mainly on the effects of white light exposure. Our systematic review incorporates the influence of all types of daytime electric light exposure (monochromatic, narrowband, polychromatic white, higher power over short-wavelength region) on alertness and higher cognitive functions. Though the use of monochromatic and narrowband light may seem impractical in real-world scenarios, we believe IIL literature is still in its exploratory stage, where much is yet to be discovered. The inclusion of all these light stimuli can help researchers to make a more informed decision while choosing their light stimuli. Additionally, to facilitate the understanding, we have clustered the studies that have reported the alpha-opic indices for the testing lights and focused on the findings of high MDER light intervention on alertness and higher cognitive functions, which is a novelty in a review of this nature.

We limit our systematic review to daytime light exposure because literature in this area is less conclusive than nocturnal IIL response (Rüger et al., 2006; Cajochen, 2007). It is fundamental to understand the extent of daytime electric light exposure’s alerting effects to facilitate the possible implications. This systematic review is timely in view of modern spectrally tuneable lighting systems that await the knowledge of the best ways to modify light to enhance alertness and higher cognitive functions.

This systematic review aims to map currently available findings on daytime electric light exposure, human alertness and higher cognitive functions. We have posed four research questions: (1) what are the effects of short-wavelength dominant daytime electric light exposure on alertness and higher cognitive functions? (2) what are the effects of the light’s intensity level on alertness and higher cognitive functions? (3) what is the potential mediation of task complexity in the relationships among light exposure, alertness and higher cognitive functions? (4) what are the effects of high MDER light exposure on alertness and higher cognitive functions?

## Methods

### Protocol and Registration

We have followed the Preferred Reporting Items for Systematic Reviews and Meta-analyses (PRISMA) statement guidelines (Moher et al., 2009) for this systematic review. The review protocol has been registered with the International Prospective Register of Systematic Reviews (PROSPERO registration number CRD42020157603).

### Eligibility Criteria

To identify studies investigating IIL responses of daytime electric light exposure on adult participants across diverse disciplines, we extend our inclusion criteria to all quantitative scholarly articles published in referred journals (Table 3). Here we focus on the studies that were performed into (a) daytime or (b) studies that compared daytime versus nighttime IIL responses. For the second group, we have reported only the results pertaining to daytime electric light exposure.

**TABLE 3:** List of inclusion-exclusion criteria.

	Inclusion criteria	Exclusion criteria
Population	Human aged 18 and above	Human aged below 18 Adults with psychiatric and physiological illness
Key area of interest	ipRGCs influenced light responses due to daytime electric light exposure on adult human	Nocturnal electric light exposure
Study design	Quantitative	Qualitative
Reporting method	Published peer-reviewed original manuscript	Editorials, conference abstracts, review articles, letters, commentaries, protocol, reports, gray literature
Outcomes	Subjective reporting of alertness, objective alertness (EEG reading, fMRI reading, eye blink test and reaction time in psychomotor vigilance task) and higher cognitive functions measured by different cognitive tasks	
Intervention: Electric light exposure	Electric light exposure of any period during daytime Electric light exposure of any period during both nighttime and daytime	Limited to nighttime/nocturnal light exposure only
Language	English	Any language other than English
Era	All years	
Field of study	All fields	
Publication status	Published research	Unpublished manuscript

### Information Sources

Before starting the database search for keywords, we commenced a database check to confirm the novelty of our systematic review in Cochrane, Prospero and PsychINFO. A robust literature search with specific keywords was conducted in PsycARTICLE via Ovid (1806–2021), PsycINFO via Ovid (1806–2021), Cochrane Library via Wiley (1973–2021), MEDLINE via Ovid (1946–2021), EMBASE and EMBASE Classic via Ovid (1947–2021), Social Science Premium Collection via ProQuest (1914–2021), ProQuest Central Via ProQuest (1970–2021), PubMed (1800–2021), Web of Science (1971–2021), Scopus (1970–2021) and Google Scholar. Finally, the forward and backward search was conducted.

### Search

Four main keywords, “light,” “cognition,” “circadian cycle” and “daytime” were analyzed to accumulate thesaurus terms (Table 4). The keywords were entered with several combinations using AND/OR. The results of each database search were documented separately. Additionally, an alert for new evidence in each database was created.

**TABLE 4:** Thesaurus analysis of keywords.

Keywords	Thesaurus analysis
Light	Radiation
	Brightness
	Illumination
	Incandescence
	Irradiation
	Luminosity
Cognition	Intelligence
	Executive functioning
	Decision making
	Attention
	Alertness
	Wakefulness
	Perception
	Memory Recall
	Recollection
Circadian cycle	Circadian Rhythm
	Biological Rhythm
	Body Clock
Daytime	Diurnal

### Study Selection

**FIGURE 3:**
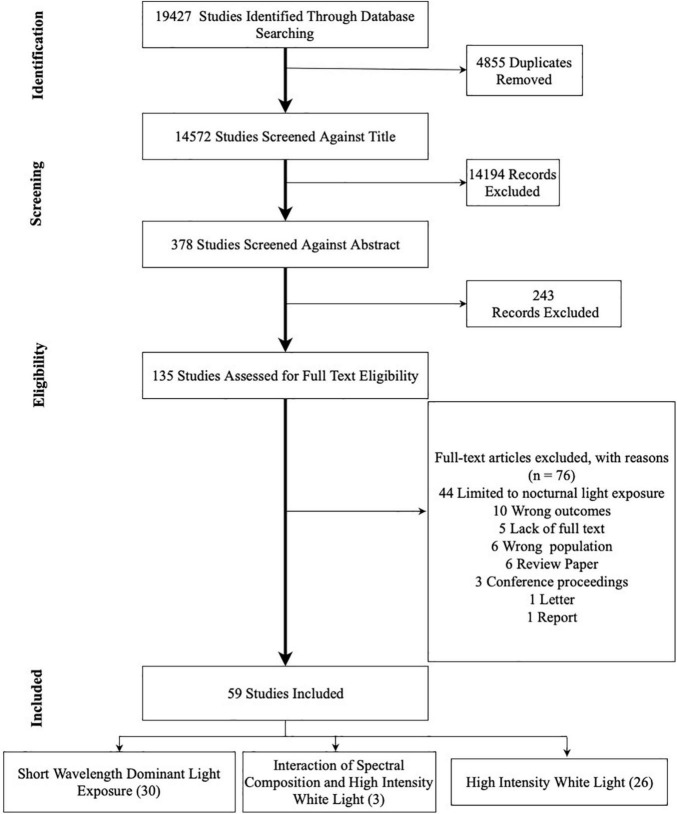
PRISMA flow chart of literature search for publication identification and selection.

MS (Mushfiqul Anwar Siraji) and SH (Shamsul Haque) conjointly completed the article selection process using Covidence^1^. The first author conducted the database searches resulting in 19,427 records (Figure 3) and imported them to Covidence. The system automatically identified 4,855 duplicate entries and removed them. Based on the eligibility criteria, both authors independently screened 14,572 records based on the titles. This title screening process further excludes 14,194 records leaving 378 records to screen against abstract. Two hundred and forty-three records were excluded, leaving 135 records for full-text review. At this stage, 76 records were excluded. For the final review, 59 articles were selected. Any disagreement between the reviewers was resolved in discussion.

### Interventions

Studies included in this systematic review focused on the effects of daytime electric light (monochromatic, narrowband, short-wavelength dominant polychromatic white) exposure of any duration on human alertness and their higher cognitive functions. We chose studies with any duration of daytime light exposure as light can activate brain regions related to alertness with as little as 2 s (Vandewalle et al., 2013). We further grouped these studies as “short-wavelength dominant light exposure” and “higher intensity white light exposure.”

### Outcomes

We checked each study on whether it reports the positive enhancing effect of daytime light exposure on subjective alertness (self-reported), objective alertness (measured by EEG, ECG, EOV, reaction time in psychomotor vigilance test-PVT and attention network component of ANT) and higher cognitive functions (accuracy and reaction time in different cognitive tasks).

### Data Extraction Process

The first author extracted the key information necessary for synthesis, including author(s) and year, participants, design, settings, study objectives, spectral and temporal characteristics of light exposure, adaptation period, exclusion and inclusion criteria for participants, methods, measures of subjective and objective alertness, measures of higher cognitive functions and data analysis. SH verified the extracted data before synthesis.

### Risk of Study Bias and Quality Assessment

Two authors (MS, SH) independently assessed the risk of study bias systematically using the methods described in the Cochrane risk of bias assessment tool (Higgins et al., 2011). Using this tool, studies were assessed to identify any potential selection bias (random sequence generation and allocation), reporting bias (selective reporting), performance bias for each outcome (blinding of the participant, personnel and outcome assessors), detection bias (blinding of outcome assessment, attrition bias) and any other sources of bias (Supplementary Figure 1). As none of the selected studies showed any potential risk of bias, we retain all 59 studies.

### Data Synthesis

**FIGURE 4:**
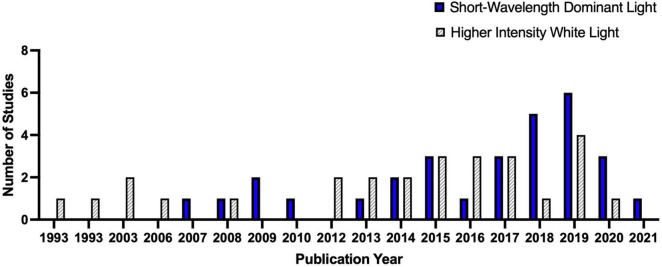
The trend of IIL literature focusing on alertness and higher cognitive functions.

We tabulated and summarized the quantitative data following the guidance from the Centre for Reviews and Dissemination (CRD, 2009). Based on the studies’ focus, we categorized them into two groups: short-wavelength-dominant electric light exposure and higher intensity white light exposure. Figure 4 depicted the trend of IIL literature focusing on alertness and higher cognitive functions. In total, we have identified 30 studies investigating the influence of short wavelength dominant light exposure and 26 studies investigating the influence of the higher intensity white light on alertness and higher cognitive functions. Only three studies investigated the interaction effect of light intensity and spectral composition on alertness and higher cognitive functions (Ru et al., 2019; Zeeuw et al., 2019; Zhu et al., 2019). We included these studies in both groups and reported only the main effects for the respective group.

We categorized the studies according to the time of day at which the light exposure started (morning: 6.00–12.00; afternoon 12.00–18.00). Further, for studies that have reported α-opic indices for their light stimuli, we have calculated the MDER following the recommendations of Schlangen and Price (2021). Subsequently, we reported the influence of higher MDER on alertness and higher cognitive functions. From the tabulated data it was observed that the included studies varied highly in terms of their sample size and intervention. Such variations in intervention and methodology made it impossible to conduct a meta-analysis.

## Results

### Controlling Interindividual Variability

Studies investigating the non-image forming effects of daytime light exposure employed several unique exclusion/inclusion criteria to minimize interindividual variability of homeostatic sleep drive, circadian cycle and sleep habits. Most studies employed a specific sleep schedule (ranging from three days to three weeks prior to the experiment; 39 studies) chronotype control (35 studies) and sleep quality assessment (20 studies) to minimize the internal circadian phase differences and match the homeostatic sleep drive of the participants.

### Effects of Electric Light Exposure on Human Alertness and Higher Cognitive Functions

Thirty-Three studies compared the effects of short-wavelength dominant light exposure on alertness and higher cognitive functions (Supplementary Table 1). Among these, 17 studies compared the influence of blue (∼470 nm) or blue-enriched white light to either amber (∼578 nm), green (∼560 nm), red (∼630 nm), or dim lights (<1–5 lux) on alertness and higher cognitive functions and another 13 compared the influence of high CCT light (4,000 K–17,000 K) to low CCT (2,500 K–4,000 K) on alertness and higher cognitive functions. Three studies compared the interaction of spectral composition and light intensity. We have reported only the main effect of spectral composition cited in these studies. The light stimuli used in these studies are also significantly different and are reported accordingly. Twenty-eight studies investigated the effect of higher intensity light exposure levels (1,000–8,121 lx) to lower intensity light exposure (10 l–500 lx) on alertness and higher cognitive functions (Supplementary Table 2).

Fourteen studies have reported α-opic indices for their test lighting conditions. Among these, only two studies (Zeeuw et al., 2019; Grant et al., 2021) have employed the new standard suggested by CIE (2018). The rest reports the α-opic indices in accordance with the CIE Technical Note 003:2015 (CIE, 2015) using toolbox provided by Lucas et al. (2014). We calculated the MDER for these studies by following the recommendations of Schlangen and Price (2021). The results are summarized in Supplementary Table 3.

#### Influence of Short Wavelength Dominant Light Exposure

**FIGURE 5:**
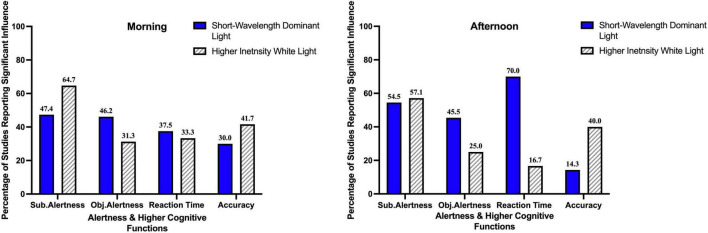
Percentage of studies reporting a significant influence of light exposure on alertness and higher cognitive functions.

Table 5 and Figure 5 summarize the findings of our current review. 47.4% (9 out of 19) studies investigating the influence of short-wavelength dominant light exposure in the morning, reported beneficial influence on subjective alertness, 46.2% (6 out of 13) reported beneficial influence on objective alertness, 37.5% (3 out of 8) reported beneficial influence on reaction time and 30% (3 out of 10) reported beneficial influence on accuracy.

**TABLE 5:** Summary of the results.

Light modification	Alerting effect				Higher cognitive functions			
	Morning Sig(total)		Afternoon Sig(total)		Morning Sig(total)		Afternoon Sig(total)	
	Sub. alertness	Objective alertness	Sub. alertness	Objective alertness	RT	ACC	RT	ACC
Short-wavelength dominant light	9 (19)	6 (13)	6 (11)	5 (11)	3 (8)	3 (10)	7 (10)	1 (7)
Higher intensity white light	11 (17)	5 (16)	8 (14)	2 (8)	3 (9)	5 (12)	1 (6)	4 (10)

54.5% (6 out of 11) the studies investigating the influence short-wavelength dominant light exposure in the afternoon, reported beneficial influence on subjective alertness, 45.5% (5 out of 11) on objective alertness, 70% (7 out of 10) on reaction time and 14.3% (1 out of 7) reported beneficial influence on accuracy.

**FIGURE 6:**
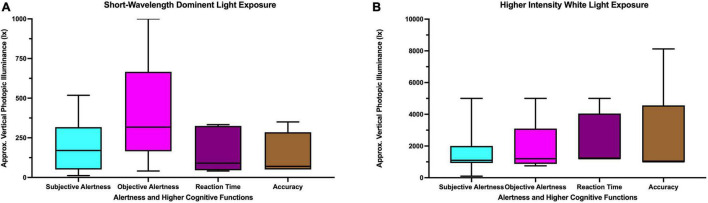
Approximate vertical photopic illuminance for studies with **(A)** short-wavelength dominant light **(B)** higher intensity white light reporting beneficial influences on alertness and higher cognitive functions.

Figure 6A summarizes the approximate vertical photopic illuminance for the studies with short-wavelength dominant light exposure reporting beneficial influences on alertness and higher cognitive functions. The photopic luminance for beneficial influences on subjective alertness ranged between 11.8 and 518 lx; for objective alertness it ranged between 40 and 1,000 lx. Beneficial influences of short-wavelength dominant light exposure were reported for reaction time with vertical photopic illuminance ranging between 40 and 333 lx. For accuracy, the vertical photopic illuminance ranged between 50 and 350 lx. The average vertical photopic illuminance for short-wavelength dominant light exposure reporting a beneficial effect on alertness and/or higher cognitive functions ranged between 135 and 395 lx.

#### Influence of Higher Intensity Bright Light

Among the studies with higher intensity bright light exposure in the morning, 64.7% (11 out of 17) reported beneficial influence on subjective alertness, 31.3% (5 out of 16) on objective alertness, 33.3% (3 out of 9) on reaction time and 41.7% (5 out of 12) reported beneficial influence on accuracy. Among the studies with higher intensity bright light exposure in the afternoon, 57.1% (8 out of 14) reported beneficial influence on subjective alertness, 25% (2 out of 8) on objective alertness, 16.7% (1 out of 6) on reaction time and 40% (4 out of 10) reported beneficial influence on accuracy.

Figure 6B summarizes the approximate vertical photopic illuminance for the studies with higher intensity white light reporting beneficial influence on alertness and higher cognitive functions. The photopic luminance for beneficial influence on subjective alertness ranged between 100 and 5,000 lx; for objective alertness it ranged between 750 and 5,000 lx. Beneficial influences of higher intensity white light were reported for reaction time with vertical photopic illuminance ranged between 1,200 and 5,000 lx. For accuracy, the vertical photopic illuminance ranged between 1,000 and 8,121 lx. The average vertical photopic illuminance for higher intensity white light exposure reporting beneficial effects on alertness and/or higher cognitive functions ranged between 1,082 and 2,424 lx.

#### Influence of High Melanopic Daylight Efficacy Ratio Light Exposure

**FIGURE 7:**
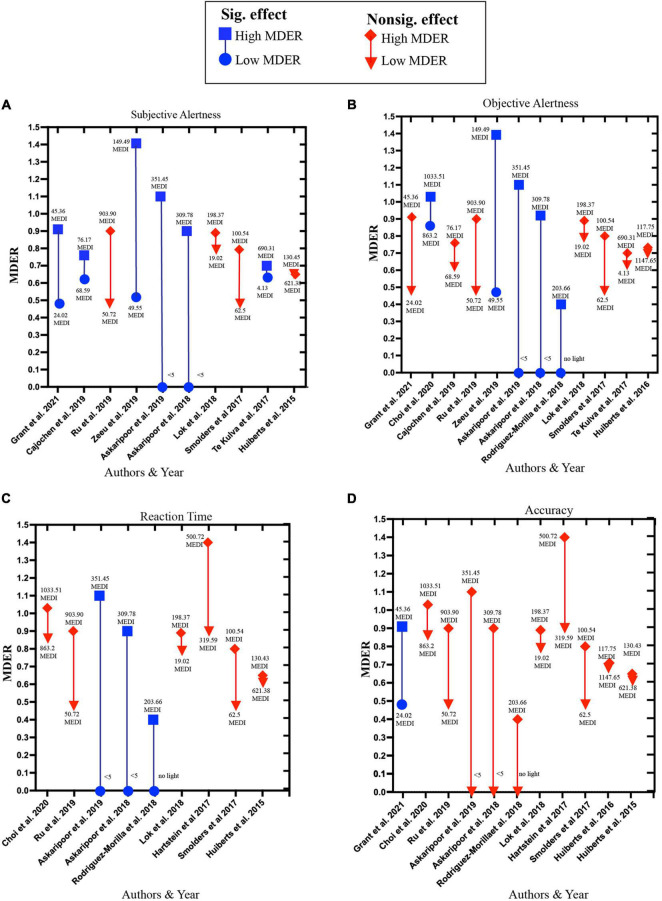
Influence of high MDER light exposure on **(A)** subjective alertness, **(B)** objective alertness, **(C)** reaction time, **(D)** accuracy. Blue squares represent high MDER light exposure in a study that reported significant beneficial effects; the corresponding blue circle represents low MDER light exposure used in the same study. The red diamond shape represents high MDER light exposure that reported insignificant effects or significant deteriorating effects. The corresponding inverted red triangle represents low MDER light exposure used in the same study.

The pattern of influence of high MDER light exposure on alertness and higher cognitive functions varies highly (Figure 7). Among the studies investigating the influence of high MDER light exposure on subjective alertness, 50% (6 out of 12) reported a beneficial influence of light exposure with MDER ranging between (0.70–1.4). For objective alertness, 41.7% of the studies (5 out of 12) reported beneficial influences of light exposure with MDER ranging between (0.4–1.4). For reaction time performance 30% of the studies (3 out of 10) reported beneficial influences of light exposure with MDER ranging between (4.3–1.1) against dim light conditions (<∼0–5 lx). For accuracy, 9% of the studies (1 out of 11) reported a beneficial influence of high MDER (0.9) light exposure on accuracy against control light (0.48).

## Discussion

The studies included in this review varied in their light modification method (short-wavelength dominant light exposure and higher intensity white light exposure), type of light (monochromatic, polychromatic, filtered), nature of exposure (continuous exposure, intermittent exposure), duration of exposure in a single session (1 s–24 h), session number (single session to 5 sessions per week) and time of day (morning and afternoon). Such variations in intervention and methodology, and the absence of the reporting of effect size in many studies made it difficult to summarize the results.

### Influence on Alertness and Higher Cognitive Functions

In general, more studies have reported alerting effects of light using subjective measures than objective measures. In the morning, 11 out of 17 (64.7%) studies with higher intensity white light exposure reported increasing subjective alertness, whereas 9 out of 19 (47.5%) studies with short-wavelength dominant light exposure reported a similar result However, in the afternoon, the percentage of reporting beneficial influence on subjective alertness is almost similar for studies with short-wavelength dominant light exposure (54.5%; 6 out of 11) and higher intensity white light exposure (57.1%; 8 out of 14). For objective alertness, a higher percentage of studies with short-wavelength dominant light exposure reported a beneficial influence both in the morning (46.2%; 6 out of 13) and in the afternoon (45.5%; 5 out of 11) compared to studies with higher intensity white light (31.3%; 5 out of 16 and 25%; 2 out of 8, respectively).

Among the studies measuring the influence of short-wavelength dominant light exposure on higher cognitive functions, a higher percentage of studies reported beneficial influences on reaction time (easy task performance) in the afternoon (70%; 7 out of 10) than morning (37.5%; 3 out of 8). However, for accuracy (complex task performance), the percentage of studies reporting a beneficial influence reduced to almost half in the afternoon (14.3%; 1 out of 17) compared to morning (30%; 3 out of 10). Among the studies measuring the influence of higher intensity white light on higher cognitive functions, a higher percentage of studies reported beneficial influence on reaction time in the morning (33.3%; 3 out of 9) than afternoon (16.7%; 1 out of 6). For accuracy, the percentage of studies reporting a beneficial effect remains almost equal for the morning (41.7%; 5 out of 12) and afternoon (40%; 4 out of 10).

The findings on beneficial influences of daytime electric light on alertness and higher cognitive functions indicate possible different mechanisms of short-wavelength dominant light exposure and high intensity white light exposure. In the afternoon, when the homeostatic sleep drive is generally higher than the morning, higher intensity white light might not contribute as much as short-wavelength dominant light toward alertness. Likewise, easy task performance (reaction time) might be benefitted more from short-wavelength dominant light exposure when our homeostatic sleep drive is high. However, further investigation is required to better understand these beneficial influences of light.

### Influenced Light Responses and Task Complexity

We found that 9 out of 22 (40%) studies reported a beneficial influence of higher intensity light exposure on accuracy (complex task performance). In contrast, only 4 out of 17 (23%) studies reported similar effects with short-wavelength dominant light exposure, regardless of when and for how long the exposure was given. This may indicate that higher intensity white light has a better ability to improve complex task performance compared to short-wavelength-dominant light exposure. However, such a conclusion cannot be firmly drawn without a meta-analysis. From the studies focusing on the higher intensity white light, it is also evident that most successful studies reporting positive influence on higher cognitive functions employed at least 1,000 lx (Figure 6). Such a high illuminance level (≥1,000 lx) has been reported to increase physiological arousal levels (Rüger et al., 2006; Smolders et al., 2012). This increased physiological arousal might play a vital role in enhancing higher cognitive functions based on the task complexity, as described by the Yerkes-Dodson Law (Yerkes, 1910). In complex task performance such as accuracy, increased performance would only appear at an optimal arousal level. This required optimal level of arousal may vary from task to task. On the other hand, easy task performance such as reaction time would linearly increase with the arousal level. Therefore, it can be suggested that higher intensity white light is required to attain a level of arousal that would be optimal for higher cognitive functions, although this mechanism may vary according to task complexity. The relationship between higher intensity white light and task complexity was explored by Huiberts et al. (2016). Their study reported an improved performance in the complex task performance for the highest intensity level (1,700 lx). However, that study failed to report any significant alerting effect. This finding could have been driven by the fact that all the light intensities (165, 600, and 1,700 lx) were already in the saturation zone of alerting effects. Such an indication of saturation is evident in the work of Cajochen et al. (2000) where a dose-dependent relationship of nighttime white light exposure and alertness was investigated and saturation of alertness was reported to start at ∼110 lx. Though no such relationship was established for daytime white light exposure, Lok et al. (2018b), in their study reported a possible saturating effect of alertness for daytime at the of 75 lx. These findings indicate a comparatively low-intensity white light is required to induce alertness and higher intensity white light is required to improve higher cognitive functions.

On the contrary, a recent study of Grant et al. (2021) has reported a beneficial influence of short-wavelength dominant light exposure on higher cognitive functions with a light intensity of 50 lx and a high MDER of.9 among moderately sleep-restricted young healthy adult. Together these findings demonstrate a plausible differential influence of short-wavelength dominant light exposure and higher intensity white light exposure on higher cognitive functions. The optimal light intensity to improve higher cognitive functions may vary depending on spectral composition (short-wavelength dominance) and task complexity.

### Importance of Spectral Composition and Illuminance

The importance of considering both the spectral composition and intensity of light can be understood from the studies employing high MDER light exposure with different light intensities (Supplementary Table 3). Among the studies reporting significant positive influence on subjective alertness, the MDER is generally high (above 70%). This pattern also holds true for objective alertness (Figure 7) with one exception (Rodriguez-Morilla et al., 2018) where a beneficial influence of light with MDER.4 is observed when compared to the no-light condition. For reaction time, it appears that having only high MDER is not sufficient. The beneficial influence on the reaction time is only reported when the high MDER experimental light exposure is coupled with comparatively higher light intensity exposure in the experimental condition than the control condition. Lastly, for accuracy only one study with high MDER light exposure (0.90) with low light intensity (50 lx) reported a beneficial influence against a control light condition with MDER (0.48) (Grant et al., 2021). Only two studies (Ru et al., 2019; Choi and Suk, 2020) employed high-intensity light exposure (lx = 1,000). However, both studies failed to report any beneficial effects on accuracy.

All these studies vary largely in terms of intensity differences between experimental conditions and control conditions. And some of the studies reported beneficial influences of light only when compared to low light (Askaripoor et al., 2018, 2019) (∼5 lx) or no light conditions (Rodriguez-Morilla et al., 2018). The current literature indicates that both short wavelength dominant light and higher intensity white light exposure induce alertness and a positive influence on higher cognitive functions. Factors that contribute toward these effects include homeostatic sleep drive, time of day, task complexity and the control light condition. However, the real magnitude of these beneficial effects remains an object of debate. Also, further investigation is required to understand the influence of short-wavelength dominant light exposure with higher intensity on alertness and higher cognitive functions.

### Methodological Issues

Since studies included in this review employed a strict exclusion-inclusion criterion to limit the interindividual variability, it is understandable that most of these studies used small samples (34.78 ± 31.70). However, such sample sizes may have led to an overall reduced statistical power in the field. Additionally, very few studies reported the use of a prior power calculation while determining the sample size. *Priori* analysis is crucial to determine the appropriate sample size to draw a precise and accurate decision (Nayak, 2010; Katherine et al., 2013; Faber and Fonseca, 2014).

Importantly, most of these studies also lacked a detailed description of the stimuli used, which creates an obstacle to potential replication attempts. Describing the stimuli only in terms of illumination, correlated color temperature, or maximum peak wavelength is insufficient (Spitschan et al., 2019; CIE, 2020). Instead, representing all this information using a spectral power distribution would provide a much clearer representation. Additionally, in the studies we have reviewed, light stimuli varied highly (monochromatic, polychromatic, filtered, fluorescent, or LED), which strengthens the need of further studies reporting the spectral radiance of the stimuli. Though the more recent studies report the spectral representation of the stimulus, only a few have reported the spectral irradiance in relation to α-opic radiances by weighting the spectrum by the spectral sensitivity of each of the five photoreceptors separately (S-, M-, L-cones, rods and ipRGCs). We strongly recommend future studies to follow the guidelines of CIE (2020) regarding how stimuli should be reported.

## Conclusion

Light affects our physiology and cognitive behavior. In this review, we focused on the beneficial influence of light on the indices of alertness and higher cognitive functions. We have categorized the studies into two groups: short-wavelength dominant light exposure and higher intensity white light exposure. We then tried to summarize the findings of these studies on our chosen outcomes. We asked four specific questions and the key findings pertaining to those four questions are summarized below:

• Both short-wavelength dominant light exposure and higher intensity light exposure can beneficially influence the indices of alertness. The pattern of influence depends on factors such as time of day and homeostatic sleep drive.
• The relationship between higher cognitive functions and light is not as straightforward as the alerting effect. The optimal level of intensity to induce improved higher cognitive performance may depend on the spectral composition, task complexity and time of day.
• A higher percentage of studies with short-wavelength dominant light exposure reported a beneficial influence on easy task performance (reaction time) in the afternoon compared to the morning. However, a reverse pattern was found for higher intensity white light.
• For complex task performance (accuracy), a higher percentage of studies reported beneficial influences of higher intensity white light on accuracy than studies with short-wavelength dominant light exposure, does not matter when and for how long the exposure was given.
• Studies with high MDER light exposure are successful in inducing alertness. However, beneficial influences are only reported for easier task performance (reaction time) when high MEDR light exposure is paired with comparatively higher intensity light. This pattern was not observed for complex task performance (accuracy).

Overall, the findings of this review indicate that the effects of daytime light exposure on alertness and higher cognitive functions are complex and dependent on various factors such as spectral composition, intensity, homeostatic sleep drive, control light condition, task complexity and time of day. Further, we found that this field of research suffered from a number of limitations, such as small sample sizes and a frequent lack of details in reporting the nature of stimuli used. Further, we also note the relative lack of studies taking place in real-life settings. Only a few studies have investigated the beneficial influence of light on alertness and higher cognitive functions in a real setting (Mills et al., 2007; Riemersma-Van Der Lek, 2008; Viola et al., 2008; Rautkylä et al., 2010; Iskra-Golec et al., 2012; Figueiro and Pedler, 2020) and most of them have limited their investigation to subjective alertness. More focused investigations considering objective alertness and especially higher cognitive functions are needed to advance the field.

## Data Availability Statement

The original contributions presented in the study are included in the article/Supplementary Material, further inquiries can be directed to the corresponding author/s.

## Author Contributions

MS, VK, AS, and SH: conceptualization. MS: data curation, formal analysis, and writing – original draft. VK and SH: supervision and writing – review and editing. AS: writing – review and editing. All authors contributed to the article and approved the submitted version.

## Conflict of Interest

The authors declare that the research was conducted in the absence of any commercial or financial relationships that could be construed as a potential conflict of interest.

## Publisher’s Note

All claims expressed in this article are solely those of the authors and do not necessarily represent those of their affiliated organizations, or those of the publisher, the editors and the reviewers. Any product that may be evaluated in this article, or claim that may be made by its manufacturer, is not guaranteed or endorsed by the publisher.

## Footnotes

1
www.covidence.org

## Supplementary Material

Supplementary Figure 1 Risk of bias summary. Green denotes low risk.
