# Supplementary figures and images for: Effects of Daytime Electric Light Exposure on Human Alertness and Higher Cognitive Functions: A Systematic Review
Source: Front Psychol. 2022 Jan 5;12:765750. doi: 10.3389/fpsyg.2021.765750 (PMC8766646; doi:10.3389/fpsyg.2021.765750)

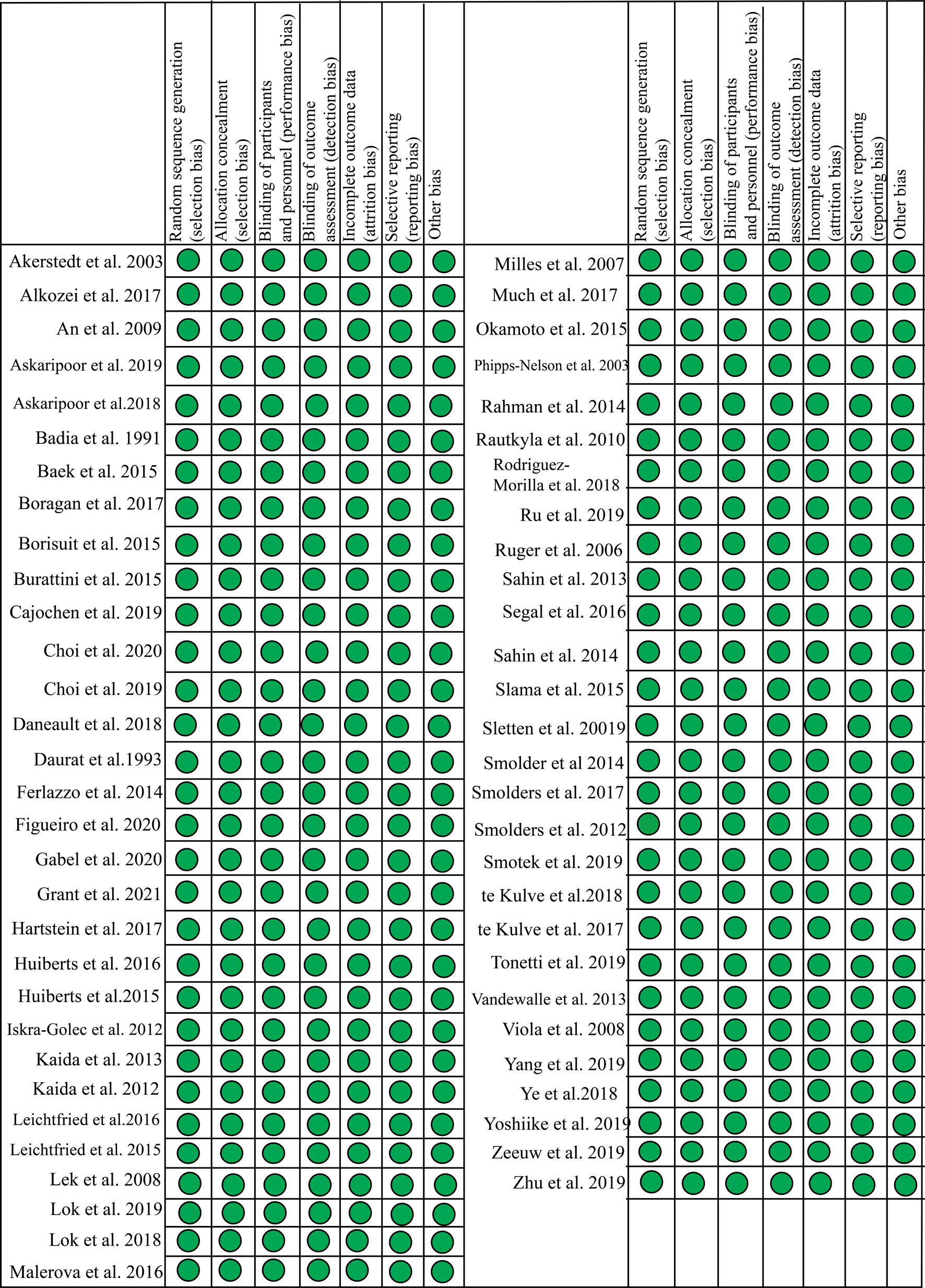

Supplement: Supplementary Figure 1 — Risk of bias summary. Green denotes low risk. [file Image_1.JPEG]
