# Supplementary material for: Effects of Daytime Electric Light Exposure on Human Alertness and Higher Cognitive Functions: A Systematic Review
Source: Front Psychol. 2022 Jan 5;12:765750. doi: 10.3389/fpsyg.2021.765750 (PMC8766646; doi:10.3389/fpsyg.2021.765750)
Supplement: Supplementary file 2 [file Table_1.docx]

**Supplementary Table 1**

*Effects of daytime short wavelength dominant electric light exposure on alertness and higher cognitive functions. "✓"indicates a significant beneficial influence of short wavelength dominant electric light exposure, ↧ indicates a deteriorate influence and "✗"indicates no significant effect.*

| **Author (year)** | **n** | **Age** | **Settings** | **Design** | **Light manipulation** | **Metrology** | | | **Light Type** | **Temporal patter** | | | **Alertness** | | | **Higher cognitive functions** | | |
| --- | --- | --- | --- | --- | --- | --- | --- | --- | --- | --- | --- | --- | --- | --- | --- | --- | --- | --- |
|  |  |  |  |  |  | **Radiometric**  **indices** | **Photometric**  **indices** | **Colorimetric condition**  **(CCT, λ_max_)** |  | **Time** | **Baseline** | **Exposure**  **Duration^1^** | **Sub. Alertness** | **Objective alertness** | | **Task** |  | |
|  |  |  |  |  |  |  | **Intensity at eye level**  **(lx)** |  |  |  |  |  |  | **Task** | **Influence** |  | **RT^2^** | **ACC.^3^** |
| Grant et al. 2021(Grant et al., 2021) | 39  (21f) | 24.5±3.2 | Lab | Between | Daylight like spectra high melEDI | 19.03 | ~50 | 5251 K | Polychromatic White LED | Morning  (2 hours after awakening) | ~3 lx | 8 h | ✓ | PVT | ✗ | Addition task |  | ✓ |
|  |  |  |  |  | Daylight like spectra low melEDI | 17.35 | ~50 | 4864 K |  |  |  |  |  |  |  |  |  |  |
|  |  |  |  |  |  |  |  |  |  |  |  |  |  |  |  | MST |  | ✓ |
|  |  |  |  |  | Conventional spectra high melEDI | 18.06 | ~50 | 3127 K |  |  |  |  |  |  |  |  |  |  |
|  |  |  |  |  | Conventional Low melEDI | 15.11 | ~50 | 2984 K |  |  |  |  |  |  |  |  |  |  |
| Figueiro et al. 2020(Figueiro & Pedler, 2020) | 70  (69f) | 39.2±11.5 | Field | Mixed | Blue Light | - | 50 | 460 nm | Wearable Lightglass  translucent  filter | Morning | - | 30 m | ↧ | PVT | ✗ | 1 back | ✗ | ✗ |
|  |  |  |  |  | Red Light | - | 50 | 630 nm |  |  |  |  |  |  |  |  |  |  |
|  |  |  |  |  | Dim White Light | - | 10 | - |  |  |  |  |  |  |  | Go-no-go | ✗ | - |
|  |  |  |  |  |  |  |  |  |  | Afternoon |  |  | ✓ | PVT | ✗ | 1 back | ✓ | ✗ |
|  |  |  |  |  |  |  |  |  |  |  |  |  |  |  |  | Go-no-go | ✓ | - |
| Choi et al. 2020(Choi & Suk, 2020) | 23  (11f) | 18.61±0.50 | Lab | Within | Controlled Scenario | - | 800-1000 | Decreasing from 6500 K-5000 K  Ra= 96 | LED lighting cabinet  970mm$\times670mm\times700 mm$ | - | 1 min | 10 m | - | EEG alpha wave | ✗ | N back | ✗ | ✗ |
|  |  |  |  |  |  |  |  |  |  |  |  |  |  | EEG beta wave | ✓ |  |  |  |
|  |  |  |  |  | Constant Scenario | - | 800-1000 | 5000 K  Ra= 98 |  |  |  |  |  |  |  |  |  |  |
|  |  |  |  |  | Light’s off scenario | - | <1 |  |  |  |  |  |  | ECG | ✓ |  |  |  |
| Cajochen et al. 2019(Cajochen et al., 2019) | 15  (0f) | 23.2±4.3 | Lab | Within | DayLED | - | 100 | 450 nm. 4000K; ra=99 | Polychromatic  White LED | Morning | 6H | 16 H | ✓ | PVT | ✗ | N-back | ✗ | ✗ |
|  |  |  |  |  | ConLED | - | 100 | Bellow 440nm,  460-520nm,  Over 620nm  4000 K;  Ra=79 |  |  |  |  |  |  |  |  |  |  |
| Choi et al.2019(Choi et al., 2019) | 15  (7f) | 23.53±0.87 | Lab | Within | Blue-enriched white light | 179.25  μW/cm^2^;  4.88 × 10^14^ photons/s cm^2^ | 518.38 | 6500K  625nm | Polychromatic  White LED | Morning  9 am | 60m  <10 lx | 60 m | ✓ | - | - | - | - | - |
|  |  |  |  |  | Warm white Light | 164.08  μW/cm^2^;  4.71 × 10^14^ photons/s cm^2^ | 516 | 3500K  460nm |  |  |  |  |  |  |  |  |  |  |
| Smotek et al. et al 2019(Šmotek, Vlček, Saifutdinova, & Kopřivová, 2019) | 12  (8f) | 17.3±2.38 |  | Within | Short | 14 μW/cm^2^ | 11.81^*^ | 455 nm | Narrow bandwidth LED | Afternoon  Post Lunch | 15mi <0.01lx | 20 m | ✓ | PVT | ✗ | - | - | - |
|  |  |  |  |  | Medium | 14 μW/cm^2^ | 15.42^*^ | 508 nm |  |  |  |  |  |  |  |  |  |  |
|  |  |  |  |  | Long | 14 μW/cm^2^ | 29.48^*^ | 629 nm |  |  |  |  |  |  |  |  |  |  |
| Tonetti et al. 2019(Tonetti & Natale, 2019) | 32 (16f) | 24.06±1.88 | Lab | Within | Blue Light | 1.9 μW/cm^2^ | 220 | 475-480 nm | Polychromatic  White LED | Afternoon  14 or 14.30 | 30 lx | 1 min | - | ANT  (alertness) | ✗ | Go-no-go | ✗ | ✗ |
|  |  |  |  |  |  |  |  |  |  |  |  |  |  |  |  | Lexical Decision task | ✗ | ✗ |
|  |  |  |  |  |  |  |  |  |  |  |  |  |  |  |  | ANT  (executive) | ✗ | ✗ |
|  |  |  |  |  | No light | - | - | - |  |  |  |  |  |  |  |  |  |  |
| Ru et al.2019(Ru et al., 2019) | 57  (38f) | 20.23±1.58 | Sim. Office  3.6 by 3.6 | Mixed | 6500 K 1000 lx | 341 μW/cm^2^  9.28 × 10^14^ photons/s cm^2^ | 1000 | 6500 K | Polychromatic White LED | Morning  10-17 | Low CCT  70 lx  High CCT 73 lx | 50 m | ✗ | PVT | ✗ | Flanker  task | ✗ | ✗ |
|  |  |  |  |  |  |  |  |  |  |  |  |  |  |  |  | PVSAT  (working memory) | ✗ | ✗ |
|  |  |  |  |  | 6500 K 100 lx | 33 μW/cm^2^  8.99 × 10^13^ photons/s cm^2^ | 100 | 6500 K |  |  |  |  |  |  |  |  |  |  |
|  |  |  |  |  | 3000 K 1000 lx | 324 μW/cm^2^  9.54 × 10^14^ photons/s cm^2^ | 1000 | 3000 K |  |  |  |  |  |  |  |  |  |  |
|  |  |  |  |  | 3000 K 100 lx | 31 μW/cm^2^  9.07 × 10^13^ photons/s cm^2^ | 100 | 3000 K |  |  |  |  |  |  |  |  |  |  |
| Zhu et al 2019(Zhu et al., 2019) | Morning 25(17f)  Afternoon 35(34f) | 22.28±0.17 | Sim. Office  4.1 $\times$ 3.3$\times2.9$ | Mixed | High Cool | - | 1200 | 6500 K  ra=82 | Polychromatic  White LED | Morning  (9-12) | 200 lx  10m | 180 m | ✗ | - | - | Go-no-go | ✗ | ↧ |
|  |  |  |  |  |  |  |  |  |  |  |  |  |  |  |  | Two back | ✗ | ✗ |
|  |  |  |  |  | High Warm | - | 1200 | 3000 K  ra=82 |  |  |  |  |  |  |  | Long term memory | ✗ | ✗ |
|  |  |  |  |  | Low Cool | - | 200 | 6500 K  ra=82 |  | Afternoon  (2:30-5.30) | 200 lx  10m | 180 m | ✗ | - | - | Go-no-go | ✗ | ↧ |
|  |  |  |  |  |  |  |  |  |  |  |  |  |  |  |  | Two back | ✗ | ✗ |
|  |  |  |  |  | Low Warm | - | 200 | 3000 K  ra=82 |  |  |  |  |  |  |  |  |  |  |
|  |  |  |  |  |  |  |  |  |  |  |  |  |  |  |  | Long term memory | ✗ | ✗ |
| Askaripoor et al 2019(Askaripoor et al., 2019) | 20  (0f) | 27.65±3.65 | Sim. Office  19m^2^ | Within | Blue enriched white light | 3.18 × 10^14^ photons/s cm^2^  121 μW/cm^2^ | 317 | 12000 K  ra=83 | Polychromatic white  Fluorescent | Afternoon  13.5 | 13 min  3520k  <5lx | 130 m | ✓ | EEG | ✓ | Continuous performance test | ✓ | - |
|  |  |  |  |  | Normal white light | 2.7 × 10^14^ photons/s cm^2^  92 μW/cm^2^ | 333 | 2700 K  ra = 81.9 |  |  |  |  |  |  |  | Two back | ✓ | - |
|  |  |  |  |  |  |  |  |  |  |  |  |  |  |  |  | Go/no-go | ✓ | - |
|  |  |  |  |  | Red saturated white light | 2.7 × 10^14^ photons/s cm^2^  96 μW/cm^2^ | 332 | 4000 K  ra= 82.9 |  |  |  |  |  |  |  |  |  |  |
|  |  |  |  |  |  |  |  |  |  |  |  |  |  |  |  | Divided Attention | ✗ | - |
|  |  |  |  |  | Dim light |  | <5 lx |  |  |  |  |  |  |  |  |  |  |  |
| Burattini et al. 2019(Burattini et al., 2019) | 40  (20f) | 22.6±1.35(f)  22.8±1.36(m) | Lab  3.6 $\times$ 2.4$\times3$ | Between group | Cold | - | 289 | 6800 | Polychromatic  White LED | Morning  10:00am-12:00pm | 15m | 15 m |  | PVT | ✓ | - | - | - |
|  |  |  |  |  | Warm | - | 288 | 3200 |  |  |  |  |  | aPVT | ✗ |  |  |  |
| Zeeuw et al. 2019(Zeeuw et al., 2019) | 72 (48f) | 24.4±2.7 | Lab | Mixed | Low illuminance Highest mel | 47.0 μW/cm^2^  1.3× 10^14^ photons/s cm^2^ | 100 | 480nm | Polychromatic  White LED | Morning  3.5 h after wakeup | 50 m  <5 lx | 180 m | ✓^b^ | AAT | ✓ ^c^ | - | - | - |
|  |  |  |  |  |  |  |  |  |  |  |  |  |  | EEG | ✓ |  |  |  |
|  |  |  |  |  | Low illuminance high mel | 41.2 μW/cm^2^  1.2× 10^14^ photons/s cm^2^ | 100 | 480nm |  |  |  |  |  |  |  |  |  |  |
|  |  |  |  |  | Low illuminance low mel | 33.1 μW/cm^2^  9.6× 10^13^ photons/s cm^2^ | 100 | 435nm |  |  |  |  |  |  |  |  |  |  |
|  |  |  |  |  | 1200 lx High mel | 528.6 μW/cm^2^  1.6× 10^15^ photons/s cm^2^ | 1200 | 480nm |  |  |  |  |  |  |  |  |  |  |
|  |  |  |  |  | 600 lx High mel | 266.2 μW/cm^2^  7.8× 10^14^ photons/s cm^2^ | 600 | 480nm |  |  |  |  |  |  |  |  |  |  |
|  |  |  |  |  | 200 lx High mel | 88.4 μW/cm^2^  2.6 × 10^14^ photons/s cm^2^ | 200 | 480nm |  |  |  |  |  |  |  |  |  |  |
|  |  |  |  |  | 1200 lx low mel | 376.4 μW/cm^2^  1.1× 10^14^ photons/s cm^2^ | 1200 | 435nm |  |  |  |  |  |  |  |  |  |  |
|  |  |  |  |  | 600 lx low mel | 187.5 μW/cm^2^  5.4× 10^14^ photons/s cm^2^ | 600 | 435nm |  |  |  |  |  |  |  |  |  |  |
|  |  |  |  |  | 200 lx low mel | 63.6 μW/cm^2^  1.8× 10^14^ photons/s cm^2^ | 200 | 435nm |  |  |  |  |  |  |  |  |  |  |
| Daneault et al. 2018(Daneault et al., 2018) | 38 | 69.6±4.9 | Lab | Within | Blue light | 3.10 × 10^13^ photons/s cm^2^  9.6 μW/cm^2^ |  | 480 nm | Polychromatic LED white light  Narrow band passes filter | Morning  3h or 5h after waking | 1m  <5lx | 40 m | ✗ | - | - | Auditory 0 back | - | ✓ |
|  |  |  |  |  | Orange monochromatic | 3.10 × 10^13^photons/s cm^2^  9.6 μW/cm^2^ |  | 620 nm |  |  |  |  |  |  |  |  |  |  |
|  |  |  |  |  |  |  |  |  |  |  |  |  |  |  |  | Auditory 2 bck | - | ✓ |
|  |  |  |  |  | Dim light |  | <5 lx |  |  |  |  |  |  |  |  |  |  |  |
| Rodriguez-Morilla et al. 2018(Rodriguez-Morilla et al., 2018) | 17  (11f) | 20.5±1.48 | lab | Within | Blue enriched white light | 4.10 × 10^14^ photons/s cm^2^  141146 μW/cm^2^ | 496 | 440nm | Polychromatic white LED light | Morning  8 am | 30m  No light | 1 hour | - | PVT | ✓ | Driving Task | ✓ | ↧ |
|  |  |  |  |  | Dim light | - | No light | - |  |  |  |  |  |  |  |  |  |  |
| Askaripoor et al 2018(Askaripoor et al., 2018) | 22  (0f) | 27.32±3.63 | Lab  4 $\times$ 5.6$\times3.05$ | Within | 7340 K | 3.14 × 10^14^ photons/s cm^2^  117 μW/cm^2^ | 333 | 7340 K  ra = 86.9 | Polychromatic white  Fluorescent light | Morning  8:30 am | 13m  5 lx  3520 K | 80 m | ✓ | EEG | ✓ | Continuous performance test | ✓ | - |
|  |  |  |  |  |  |  |  |  |  |  |  |  |  | Heart rate | ✓ |  |  |  |
|  |  |  |  |  | 3730 K | 2.7 × 10^14^ photons/s cm^2^  96 μW/cm^2^ | 332 | 3730 K  ra = 82.9 |  |  |  |  |  |  |  |  |  |  |
|  |  |  |  |  |  |  |  |  |  | Afternoon  1:50  pm | 13m  5 lx  3520 K | 80 m | ✓ | EEG | ✓ | Continuous performance test | ✓ | - |
|  |  |  |  |  | 2564 K | 2.7 × 10^14^ photons/s cm^2^  92 μW/cm^2^ | 333 | 2564 K  ra = 81.9 |  |  |  |  |  |  |  |  |  |  |
|  |  |  |  |  |  |  |  |  |  |  |  |  |  | Heart rate | ✓ |  |  |  |
|  |  |  |  |  | Dim light | - | <5 |  |  |  |  |  |  |  |  |  |  |  |
| Te Kulve et al 2018(te Kulve et al., 2018) | 16  (0f) | 22.2±2.37 | Lab | Within | 6500 K |  | 55 | 6500 K | Polychromatic white LED light | Morning  8:30 am | 45m  5 lx  4000km | 75 m | ✓ | PVT | ✗ | - | - | - |
|  |  |  |  |  | 2700 K |  | 55 | 2700 K |  |  |  |  |  |  |  |  |  |  |
| Ye et al 2018(Ye et al., 2018) | 10  (3f) | 23±1.5 | Lab | Within | High CCT Range | - | 500 | 6000-12000 K | Dynamic LED | Afternoon  12:30 pm | 30 m  <10 lx | 270 m | ✓ | EEG | ✓ | Go-no-go | ✗ | ✗ |
|  |  |  |  |  | Low CCT  Range | - | 500 | 4000-1000 K |  |  |  |  |  |  |  | 2 back | ✓ | - |
|  |  |  |  |  |  |  |  |  |  |  |  |  |  |  |  | 3 back | ✓ | - |
|  |  |  |  |  |  |  |  |  |  |  |  |  |  |  |  | MATB-II | - | ✗ |
| Alkozei et al 2017(Alkozei et al., 2017) | 30  (17f) | 21.87±3.74 | Lab | Between | Blue Light | 0.11 μW/cm^2^ | 214 | 469 nm | Monochromatic  Narrow Bandwidth Fluorescent | Morning  9 am | 30 m | 30 m | - | - | - | California Verbal Learning Test II |  | ✓ |
|  |  |  |  |  | Amber | 0.04 μW/cm^2^ | 188 | 578 nm |  |  |  |  |  |  |  |  |  |  |
| Hartstein et al 2017 | 40  (21f) | 18-26 | Lab  2.6 m$\times2.2 m$ | Mixed | Cool light | - | 350 | 5000 K | Polychromatic white Light | Morning  (9:00 am or 10:00 am) | 20 m | - | - | - | - | Go-no go | ✓^d^ | ✗ |
|  |  |  |  |  | Warm Light | - | 350 | 3500 K |  |  |  |  |  |  |  | Task Switching | ✗ | ✗ |
| Smolder et al 2017(Smolders & de Kort, 2017) | 39  (12f) | 23±3.9 | Simulated office  3.9 m$\times3.7 m$ | Mixed | 6000 K | 1.11 × 10^14^ photons/s cm^2^  41 μW/cm^2^ | 124 | 6000 K | Polychromatic white LED light | Morning  9 am /11 am | 158 lx  4000 K | 60 m | ✗ | Heart rate | ↧ | Addition task | - | ✗ |
|  |  |  |  |  |  |  |  |  |  |  |  |  |  |  |  | Letter  cancellation task | - | ↧ |
|  |  |  |  |  |  |  |  |  |  |  |  |  |  | Skin Conductance | ✗ |  |  |  |
|  |  |  |  |  | 2700 K | 1.11 × 10^14^ photons/s cm^2^  39 μW/cm^2^ | 132 | 2700 K |  |  |  |  |  |  |  |  |  |  |
|  |  |  |  |  |  |  |  |  |  |  |  |  |  | ANT  (attentat) | ✗ | ANT  (executive) | ✗ | ✗ |
|  |  |  |  |  |  |  |  |  |  | Afternoon  1 p, / 3 pm | 158 lx  4000 K | 60 m | ✗ | Heart rate | ✗ | Addition task |  | ↧ |
|  |  |  |  |  |  |  |  |  |  |  |  |  |  |  |  | Letter  cancellation task | - | ✗ |
|  |  |  |  |  |  |  |  |  |  |  |  |  |  | Skin Conductance | ✗ |  |  |  |
|  |  |  |  |  |  |  |  |  |  |  |  |  |  |  |  | ANT  (executive) | ✗ | ✗ |
|  |  |  |  |  |  |  |  |  |  |  |  |  |  | ANT  (attentat) | ✗ |  |  |  |
| Segal et al 2016(Segal, Sletten, Flynn-Evans, Lockley, & Rajaratnam, 2016) | 60  (31f) | 18-31 | Lab | Between | Blue | 2.8 × 10^13^ photons/s cm^2^  13.45 μW/cm^2^ | - | 458 nm | Monochromatic  Narrow Bandwidth Fluorescent | Morning  2.5 h after waking up | <3 lx | 180 m | ✗ | aPVT | ✗ | Stroop | ✗ | - |
|  |  |  |  |  |  |  |  |  |  |  |  |  |  |  |  | 2-back Test | ✗ | - |
|  |  |  |  |  |  |  |  |  |  |  |  |  |  | EEG | ✗ |  |  |  |
|  |  |  |  |  | Green | 2.8 × 10^13^ photons/s cm^2^  11.01 μW/cm^2^ | - | 551 nm |  |  |  |  |  |  |  |  |  |  |
| Baek et al 2015(Baek & Min, 2015) | 20  (11F) | 24.5 | Lab | Within | 66% Blue Enriched | - | 40.6^*^ | 451 nm | Polychromatic White Lights  LED | Afternoon  2:00 pm | <0.3 lx  10 m | 60 m | - | EEG | ✓ | Continuous Performance Test | ✓ | ✗ |
|  |  |  |  |  | 33% Blue Enriched | - | 40.1^*^ | 451 nm |  |  |  |  |  |  |  |  |  |  |
|  |  |  |  |  | White Light | - | 40.2^*^ | - |  |  |  |  |  |  |  |  |  |  |
|  |  |  |  |  | Dark | - | <.3 lx | - |  |  |  |  |  |  |  |  |  |  |
| Okamoto et al 2015(Okamoto & Nakagawa, 2015) | 8  (0f) | 22.9 | Lab | Within | Short Wavelength | 33.9 × 10^12^ photons/s cm^2^  14.4 μW/cm^2^ | 10 | 470 nm | Monochromatic LED Light | Afternoon  12:00 | Dark  10 m | 28 m | ✗ | EEG | ✓ | Auditory odd ball | ✓ | - |
|  |  |  |  |  | Medium Wavelength | 4.9 × 10^12^ photons/s cm^2^  1.8 μW/cm^2^ | 10 | 530 nm |  |  |  |  |  |  |  |  |  |  |
|  |  |  |  |  | Long Wavelength | 14.9 × 10^12^ photons/s cm^2^  4.7 μW/cm^2^ | 10 | 620 nm |  |  |  |  |  |  |  |  |  |  |
|  |  |  |  |  | Dark |  | 0.01 |  |  |  |  |  |  |  |  |  |  |  |
| Slama el al 2015(Slama, Deliens, Schmitz, Peigneux, & Leproult, 2015) | 10  (8f) | 23.4±1.6 | Lab | Mixed | Blue enriched white light |  | 2000 | 460 nm | Lumiette | Afternoon  (After lunch) | 300 lx | 30 m | ✗ | PVT | ✗ | Task switching | ✓ | ✓ |
|  |  |  |  |  | Orange enriched white light |  | <200 | 600 nm |  |  |  |  |  |  |  |  |  |  |
|  |  |  |  |  | Darkness |  | 0 | - |  |  |  |  |  |  |  |  |  |  |
| Rahman et al 2014(Rahman et al., 2014) | 9  (0f) | 24.8±2.6 | Lab | Between | 460 nm | 2.8 × 10^13^ photons/s cm^2^  12.1 μW/cm^2^ | - | 460 nm | Xenon arc lamp with  grating monochromator filter | Morning  4.75 h after wake up | ~1.5 lx | 390 m | ✗ | aPVT | ✓ | - | - | - |
|  |  |  |  |  |  |  |  |  |  |  |  |  |  | EEG | ✓ |  |  |  |
|  |  |  |  |  | 555 nm | 2.8 × 10^13^ photons/s cm^2^  10 μW/cm^2^ | - | 555 nm |  |  |  |  |  |  |  |  |  |  |
| Ferlazzo et al 2014(Ferlazzo et al., 2014) | 44  (22f) | F: 25.6±3.87  M: 25.31±4.85 | Lab  3.6m $\times2.4 m\times3 m$ | Between | LED | 27.62  μW/cm^2^  276.2 lx | 90 | 4000 K, ra= 80 | Polychromatic white LED | - | 2800 K  655.9 lx | 20 m | - | - | - | Purdue Visualization Rotation | ✗ | ✓ |
|  |  |  |  |  | Halogen | 65.59  μW/cm^2^  655.9 lx | 100 | 2800 K, ra= 100 | Halogen |  |  |  |  |  |  |  |  |  |
|  |  |  |  |  |  |  |  |  |  |  |  |  |  |  |  | Task Switching paradigm | ✓ | ✗ |
| Sahin et al 2013(Sahin & Figueiro, 2013) | 13  (5f) | M:20.5  F: 21 | Lab | Within | Blue | 18.9 μW/cm^2^ | 40 | 470 nm | LED with full width at half maximum 25 nm | Afternoon  2: 30 pm | 12m  <0.01 lx | 48 m | ✗ | EEG | ✗ | - | - | - |
|  |  |  |  |  | Red | 40.2 μW/cm^2^ | 40 | 630 nm |  |  |  |  |  |  |  |  |  |  |
|  |  |  |  |  | Dark | - | <0.01 |  |  |  |  |  |  |  |  |  |  |  |
| Iskra-Golec et al 2012(Iskra-Golec et al., 2012) | 30  (all f) | 28.3±2.8 | Field:  Airline office | Within | Blue enriched white light | - | 500 | 17000 K  ra= 82 | Polychromatic white  FL | Moring  (7:15) | - | 8 h | ✗ | - | - | - | - | - |
|  |  |  |  |  | Neutral white light | - | 500 | 4000 K  Ra=85 |  |  |  |  |  |  |  |  |  |  |
| Rautkyla et al 2010(Rautkylä et al.) | Spring : 16 (0f)  Autumn: 138 (17f) | S:24±1.3  A: 22±1.8 | Classroom  620m^2^ | Within | 17000 K |  | 800-1000 | 17000 K  ra> 80 | Polychromatic white  Fluorescent | Morning  8.15/9.15 am | - | 90 m | ✗ | - | - | - | - | - |
|  |  |  |  |  | 4000 K |  | 800-1000 | 4000 K  ra> 80 |  |  |  |  |  |  |  |  |  |  |
|  |  |  |  |  |  |  |  |  |  | Afternoon  12.15/2.15 pm | - | 90 m | ✓^e^ | - | - | - | - | - |
| An et al 2009(An, Huang, Shimomura, & Katsuura, 2009) | 12  (0f) | 20.92±1.08 | Lab | Within | 458 nm | 9.8 μW/cm^2^ |  | 458 nm | Halogen lamp with 10 nm interference filters | Daytime | 5 m  <1 lx | 28 m | ✗ | Alpha attenuation coefficient | ✗ | - | - | - |
|  |  |  |  |  | 550 nm | 9.8 μW/cm^2^ |  | 550 nm |  |  |  |  |  |  |  |  |  |  |
| Sletten et al 2009(Sletten, Revell, Middleton, Lederle, & Skene, 2009) | Young 11  Older: 15 | Y: 23±2.9  O: 65.8±5.0 | Lab | Within | 456 nm | 26.4 μW/cm^2^  6.04 × 10^13^ photons/s cm^2^ |  | 456 nm | Mercury and metal Halide arc lamp with narrow bandwidth interference filter | Morning  8.5 after DLMO^f^ | <10 lx | 120 m | ✓ | - | - | - | - | - |
|  |  |  |  |  | 548 nm | 21.7 μW/cm^2^  5.93 × 10^13^ photons/s cm^2^ |  | 548 nm |  |  |  |  |  |  |  |  |  |  |
| Viola et al 2008(Viola et al., 2008) | 94 |  | Two real office floor | Within | Blue enriched white light | 2.58-6.42 × 10^14^  μW/cm^2^ | 310.35^a^ | 17000 K | Polychromatic white  Fluorescent | Morning  8:30 am | - | 8 h | ✓ | - | - | - | - | - |
|  |  |  |  |  | White light | 2.4-4.49 × 10^14^  μW/cm^2^ | 421.07^a^ | 4000 K |  |  |  |  |  |  |  |  |  |  |
| Mills et al 2007(Mills et al., 2007) | 69 |  | Real office floor |  | 17000 K |  | 170 | 17000 K | Polychromatic white  FL | Morning  8 am | 2900 K | 12 h | ✓ | - | - | - | - | - |
|  |  |  |  |  | 2900 K |  | 128 | 2900 K |  |  |  |  |  |  |  |  |  |  |

Note. ^*^Horizontal Lux

^1^exposure duration in a single session

^2^ RT = reaction time

^2^Acc =Accuracy

^a^Measured at the desk level

^b^Twenty minutes after the light exposure

^c^For low illuminance highest mel-lighting condition

^d^For Males only

^e^For autumn study.

^f^DLMO: Dim light melatonin onset
