# Supplementary material for: Effects of Daytime Electric Light Exposure on Human Alertness and Higher Cognitive Functions: A Systematic Review
Source: Front Psychol. 2022 Jan 5;12:765750. doi: 10.3389/fpsyg.2021.765750 (PMC8766646; doi:10.3389/fpsyg.2021.765750)
Supplement: Supplementary file 3 [file Table_2.docx]

**Supplementary Table 2**

*Effects of daytime polychromatic higher intensity white light exposure on alertness and higher cognitive functions.*

*"✓"indicates a significant beneficial influence of bright light in comparison to control light condition is reported, ↧ indicates a deteriorate influence is reported and "✗"indicates no significant effect is reported.*

|  | **Author (year)** | **n** | **Age** | **Settings** | **Design** | **Light manipulation** | **Metrology** | | | | **Light type** | **Temporal patter** | | | **Alertness** | | | **Higher cognitive functions** | | | |
| --- | --- | --- | --- | --- | --- | --- | --- | --- | --- | --- | --- | --- | --- | --- | --- | --- | --- | --- | --- | --- | --- |
|  |  |  |  |  |  |  | **Radiometric**  **indices** | **Photometric**  **indices** | **Colorimetric condition** | |  | **Time** | **Baseline** | **Exposure**  **Duration^1^** | **Sub. Alertness** | **Objective alertness** | | **Task** | | **Performance** | |
|  |  |  |  |  |  |  |  | **Intensity at eye level**  **(lx)** | **CCT (K)/λmax** | **Ra** |  |  |  |  |  | **Task** | **RT** |  |  | **RT** | **ACC** |
| Gabel et al. 2020(Gabel, Miglis, & Zeitzer, 2020) | | 23 (13F) | 67.3±8.8 | Lab | Within | Dawn  Simulation | (1.4E+17 -  2.4E+18) photons/s m^2^ |  | 1090 -2750 K |  | Polychromatic white | Morning  Half an hour prior  To waking up | <0.05 lx | 30 m | ✗ | PVT | ✗ | - | | - | - |
|  |  |  |  |  |  | Dark |  | <0.05 lx |  |  |  |  |  |  |  | HR | ✓ |  |  |  |  |
| Zhu et al. 2019(Zhu et al., 2019) | | 60  (51f) | 22.28±0.17 | Simulated window less office  4.1$m\times3.3m\times2.9m$ | Mixed | High Cool | - | 1200 | 6500 K | 82 | Polychromatic white  LED | Morning  (9-12) | 200 lx  10 m | 180 m | ✓ | - | - | Go-no-go | | ✓ | ✗ |
|  |  |  |  |  |  |  |  |  |  |  |  |  |  |  |  |  |  | 2-back | | ✓ | ✓ |
|  |  |  |  |  |  | High Warm | - | 1200 | 3000 K | 83 |  |  |  |  |  |  |  | Long term memory | | ✗ | ✗ |
|  |  |  |  |  |  | Low Cool | - | 200 | 6500 K | 82 |  |  |  |  |  |  |  |  |  |  |  |
|  |  |  |  |  |  | Low Warm | - | 200 | 3000 K | 82 |  |  |  |  |  |  |  |  |  |  |  |
|  |  |  |  |  |  |  |  |  |  |  |  | Afternoon  (2.30-5.30) |  | 180 m | ✓ | - | - | Go-no-go | | ✓ | ✗ |
|  |  |  |  |  |  |  |  |  |  |  |  |  |  |  |  |  |  | 2-back | | ✓ | ✓ |
|  |  |  |  |  |  |  |  |  |  |  |  |  |  |  |  |  |  | Long term memory | | ✗ | ✓ |
| Ru et al. 2019(Ru et al., 2019) | | 57 (38f) | 20.23±1.58 | Simulated office  3.6 by 3.6 m | Mixed | 3000 K 1000 lx | 324 μW/cm^2^;  9.54 × 10^14^ photons/s cm^2^ | 1000 | 3000 K | 83 | Polychromatic white  LED | Morning  (10:00-17:00) | 70-73 lx | 50 m | ✗ | PVT | ✗ | Go-no-go | | ✓ | ✗ |
|  |  |  |  |  |  | 3000 K 100 lx | 31 μW/cm^2^;  9.07 × 10^13^ photons/s cm^2^ | 100 | 3000 K | 83 |  |  |  |  |  |  |  | Flanker test | | ✗ | ✗ |
|  |  |  |  |  |  | 6500 K 1000 lx | 331 μW/cm^2^;  9.28× 10^14^ photons/s cm^2^ | 1000 | 6500 K | 81 |  |  |  |  |  |  |  | PVSAT | | ✗ | ✓ |
|  |  |  |  |  |  | 6500 K 100 lx | 33 μW/cm^2^;  8.99× 10^13^ photons/s cm^2^ | 100 | 6500 K | 81 |  |  |  |  |  |  |  |  |  |  |  |
| Zeeuw et al. 2019(Zeeuw et al., 2019) | | 72  (48f) | 24.4±2.7 | Lab | Mixed | 12000 lx High mel | 528.6 μW/cm^2^  1.6× 10^15^ photons/s cm^2^ | - | 480nm |  | Polychromatic white  LED | Morning  3.5 H after wakeup | 50 m  <5 lx | 180 m | ✗ | AAT Index | ✗ | - | | - | - |
|  |  |  |  |  |  |  |  |  |  |  |  |  |  |  |  | EEG | ✓ |  |  |  |  |
|  |  |  |  |  |  | 12000 lx low mel | 376.4 μW/cm^2^  1.1× 10^14^ photons/s cm^2^ | - | 435nm |  |  |  |  |  |  |  |  |  |  |  |  |
|  |  |  |  |  |  | 600 lx High mel | 266.2 μW/cm^2^  7.8× 10^14^ photons/s cm^2^ | - | 480nm |  |  |  |  |  |  |  |  |  |  |  |  |
|  |  |  |  |  |  | 600 lx low mel | 187.5 μW/cm^2^  5.4× 10^14^ photons/s cm^2^ | - | 435nm |  |  |  |  |  |  |  |  |  |  |  |  |
|  |  |  |  |  |  | 200 lx High mel | 88.4 μW/cm^2^  2.6 × 10^14^ photons/s cm^2^ | - | 480nm |  |  |  |  |  |  |  |  |  |  |  |  |
|  |  |  |  |  |  | 200 lx low mel | 63.6 μW/cm^2^  1.8× 10^14^ photons/s cm^2^ | - | 435nm |  |  |  |  |  |  |  |  |  |  |  |  |
|  |  |  |  |  |  | Low illuminance Highest mel | 47.0 μW/cm^2^  1.3× 10^14^ photons/s cm^2^ | - | 480nm |  |  |  |  |  |  |  |  |  |  |  |  |
|  |  |  |  |  |  | Low illuminance high mel | 41.2 μW/cm^2^  1.2× 10^14^ photons/s cm^2^ | - | 480nm |  |  |  |  |  |  |  |  |  |  |  |  |
|  |  |  |  |  |  | Low illuminance low mel | 33.1 μW/cm^2^  9.6× 10^13^ photons/s cm^2^ | - | 435nm |  |  |  |  |  |  |  |  |  |  |  |  |
|  |  |  |  |  |  | Dim light |  |  |  |  |  |  |  |  |  |  |  |  |  |  |  |
| Yoshike et al.  2019(Yoshiike, Honma, Ikeda, & Kuriyama, 2019) | | 24  (10f) | 21.8±1.3 | Lab | Between  group | Bright light | - | 8121 | 6700 K |  | Polychromatic white  LED | Afternoon  (1:00 PM) |  | 15 m | ✗ | - | - | Motor skill performance | | ✗ | ✓ |
|  |  |  |  |  |  | Control light | - | 429 | 6700 K |  |  |  |  |  |  |  |  |  |  |  |  |
| Yang et al.  2019(Yang et al., 2019) | | 59  (35f) | 20.3±2.1 (MT)  20.4±1.7  (ET) | Simulated Office  4.1m$\times$3.3m$\times$2.0m | Mixed | 1200 lx | 17.58 × 10^13^ photons/s cm^2^  3.99 W/cm^2^ | 1200 | 6700 K | 82 | Polychromatic white  LED | Morning  (1.5 h after wakening up) | 12m  100 lx | 46 m | ✓ | aPVT | ✗ | Task  Switching | | ✓ |  |
|  |  |  |  |  |  | 200 lx | 3.05 × 10^13^ photons/s cm^2^  0.69W/cm^2^ | 200 | 6700 K | 82 |  |  |  |  |  |  |  |  |  |  |  |
| Lok et al.  2019(Lok, van Koningsveld, Gordijn, Beersma, & Hut, 2019) | | 10  (5f) | 20-30 | Lab | Within | Bright Light | - | 2000 | - | - | Polychromatic white  LED | Afternoon  (2.30-4.00) | 10 lx,  1 h | 90 m | ✗ | aPVT | ✗ | - | | - | - |
|  |  |  |  |  |  | Dim Light | - | 10 | - | - |  |  |  |  |  |  |  |  |  |  |  |
| Lok et al.  2018(Lok, Woelders, et al., 2018) | | 50  (25f) | 23.02±0.29 | Lab | Mixed | 666 lx | - | - | - | - | Polychromatic white  LED | Morning  (7:30 -17:30) | <10 lx | 60 m | ✓ | Eye Blink Test | ↧ | Go-no-go | | ✗ | ↧ |
|  |  |  |  |  |  | 222 lx | - | - | - | - |  |  |  |  |  |  |  |  |  |  |  |
|  |  |  |  |  |  | 74 lx | - | - | - | - |  |  |  |  |  |  |  |  |  |  |  |
|  |  |  |  |  |  | 24 lx | - | - | - | - |  |  |  |  |  |  |  |  |  |  |  |
| Munch et al. 2017(Munch et al., 2017) | | 18 (12f) | 23.2±3.3 | Lab | Mixed | Mixed  blue enriched lighting | - | 750 | 3537 K |  | Polychromatic white  LED & FL | Morning  8 am -11 am | - | 3 h | ✓ | PVT | ✓ | - | - | - | - |
|  |  |  |  |  |  | Bright blue light | - | 500 | 6500 K |  |  |  |  |  |  |  |  |  |  |  |  |
|  |  |  |  |  |  | Orange light | - | 100 | 1500 K |  |  |  |  |  |  |  |  |  |  |  |  |
|  |  |  |  |  |  | Control light | - | 40 | 2600 K |  |  |  |  |  |  |  |  |  |  |  |  |
|  |  |  |  |  |  | Dim light | - | <7 | 2700 K |  |  |  |  |  |  |  |  |  |  |  |  |
| Te Kulve et al.  2017(te Kulve, Schlangen, Schellen, Frijns, & Lichtenbelt, 2017) | | 19  (All f) | 22.3±1.9 | Lab | Mixed | Bright light | - | 986 | 4000 K | - | Polychromatic white  LED | Morning  (08:30 AM-01:00PM) | 250 lx  4000 K  250 lx | 75 m | ✓ | aPVT | ✗ | - | - | - | - |
|  |  |  |  |  |  | Dim light | - | 4.13 | 4000 K | - |  |  |  |  |  |  |  |  |  |  |  |
| Borragan et al. 2017(Boubekri et al., 2020) | | 20(8f) | 23.7±3.6 | Lab | Within | Bright light |  | 2000 |  |  | Wearable light glass | Afternoon  (9 h after awaking) |  | 20 m | ✗ | PVT | ✗ | TloadDback | | - | ✗ |
|  |  |  |  |  |  | Dim light |  | <200 |  |  |  |  |  |  |  |  |  |  |  |  |  |
| Maierova et al.  2016(Maierova et al., 2016) | | 32  (18f) | 22.7±3.5 | Lab | Within | Bright Light | - | 1000 | - | - | Polychromatic white | Morning  (one hour after habitual wake time |  | 16 h | ✓ | aPVT | ✗ | Auditory 2 back | | ✗ | ✗ |
|  |  |  |  |  |  | Self-selected | - | Up to 1200 lx | - | - |  |  |  |  |  |  |  | Long term memory | | ✗ | ✗ |
|  |  |  |  |  |  | Dim light | - | <5 | - | - |  |  |  |  |  |  |  | Visual 2 back | | - | ✓ |
|  |  |  |  |  |  |  |  |  |  |  |  |  |  |  |  |  |  | Visual 3 back | | - | ✓ |
|  |  |  |  |  |  |  |  |  |  |  |  |  |  |  |  |  |  | Go-no-go | | - | ✗ |
| Huiberts et al.  2016(Huiberts et al., 2016) | | 39  (28f) | 21.2±2.1 | Simulated office  3.9 m $\times$ 7.4 m | Mixed | 1700 lx | 1.39 × 10^15 photons/s cm^2^  530 μW/cm^2^ | 1700 | 4700 K | 87 | Polychromatic white  FL | Morning  (9-10.30) | 120 lx | 60 m | ✗ | aPVT | ✗ | Backward digit span test | | - | ✓ |
|  |  |  |  |  |  | 600 lx | 4.96 × 10^14 photons/s cm^2^  188 μW/cm^2^ | 600 | 4700 K | 87 |  |  |  |  |  |  |  |  |  |  |  |
|  |  |  |  |  |  | 165 lx | 1.34 × 10^14 photons/s cm^2^  51 μW/cm^2^ | 165 | 4700 K | 87 |  |  |  |  |  |  |  |  |  |  |  |
|  |  |  |  |  |  |  |  |  |  |  |  | Afternoon  (15:45-17:15) |  | 60 m | ✗ | aPVT | ✗ | Backward digit span test | |  | ✓ |
| Leichtfried et al. 2016(Leichtfried, Hanser, Griesmacher, Canazei, & Schobersberger, 2016) | | 15(9) | 31.3±8.5 | Lab | Within | Bright light | - | 5000 | 6500 K | - | Polychromatic white  FL | Morning  7:40 am |  | 30 | - | Sustained attention | ✓ | Sport specific performance | | - | ✗ |
|  |  |  |  |  |  | Dim light | - | 150 | 4000 K | - |  |  |  |  |  |  |  |  |  |  |  |
| Leichtfried et al. 2015(Leichtfried et al., 2015) | | 33 (17f) | 33.0±7.2 | Lab | Within | Bright light | - | 5000 | 6500 K | - | Polychromatic white  FL | Morning  7:40 am |  | 30 | ✓ | Sustained attention | ↧ | - | | - | - |
|  |  |  |  |  |  | Dim light | - | 400 | 4000 K | - |  |  |  |  |  |  |  |  |  |  |  |
| Borisuit et al.  2015(Borisuit, Linhart, Scartezzini, & Münch, 2015) | | 25  (9f) | 23.5±2.3 | Lab  7 m $\times$ 5 m | Mixed | Day light condition | 3.5-7.0 μW/cm2 | 1000-2000 | 4420-4701 K | 89.9-95.4 | Polychromatic white  FL | Afternoon  (12:00 PM-8PM) | - | 5 h | ✓ | - | - |  | |  |  |
|  |  |  |  |  |  | Electric Light | 0.5 μW/cm^2^ | 173.6 | 4000 K | 83.4 |  |  |  |  |  |  |  |  |  |  |  |
| Huiberts et al.  2015(Huiberts et al., 2015) | | 64  (32f) | 21.4±2.1 | Lab | Mixed | 1000 lx | 8.09 × 10^14 photons/s cm^2^  304 μW/cm^2^ | 1000 | 4000 K | 87 | Polychromatic white  FL | Morning | 100 lx  4000 K  7.88$\times$10^13^  photons/cm^2^/s  30 μW/cm^2^  15 min | 60 m | ✓ | - | - | Forward  Digit Span | |  | ✓ |
|  |  |  |  |  |  | 200 lx | 1.63 × 10^14 photons/s cm^2^  61μW/cm^2^ | 200 | 4000 K | 87 |  |  |  |  |  |  |  | Backward digit span | |  | ✓ |
|  |  |  |  |  |  |  |  |  |  |  |  |  |  |  |  |  |  | 1 back | | ✗ | ✗ |
|  |  |  |  |  |  |  |  |  |  |  |  |  |  |  |  |  |  | 2 back | | ✗ | ✗ |
|  |  |  |  |  |  |  |  |  |  |  |  |  |  |  |  |  |  | 113 back | | ✗ | ✗ |
|  |  |  |  |  |  |  |  |  |  |  |  | Afternoon |  | 60 m | ✗ | - | - | Forward  Digit Span | |  | ✓ |
|  |  |  |  |  |  |  |  |  |  |  |  |  |  |  |  |  |  | Backward digit span | |  | ↧ |
|  |  |  |  |  |  |  |  |  |  |  |  |  |  |  |  |  |  | 1 back | | ✗ | ✗ |
|  |  |  |  |  |  |  |  |  |  |  |  |  |  |  |  |  |  | 2 back | | ✗ | ↧ |
|  |  |  |  |  |  |  |  |  |  |  |  |  |  |  |  |  |  | 3 back | | ✗ | ✗ |
| Sahin et al. 2014(Sahin, Wood, Plitnick, & Figueiro, 2014) | | 13 (7f) | 23±5.5 | Lab | Within | Red light | 1.1 W/m2 | 213 lx | - |  | Red Led | Morning7:00 Midday 11:00 Afternoon 15:00 | <5 lx  10 min | 110 m | ✗ | EEG | ✗ | Go no go | | ✗ |  |
|  |  |  |  |  |  | White light | 1.1 W/m2 | 361 lx | 2568±22 K |  | Polychromatic white  LED |  |  |  |  |  |  | Multi attribute task | |  | ↧ |
|  |  |  |  |  |  | Dim light |  | <5 lx | - |  |  |  |  |  |  |  |  |  |  |  |  |
| Smolders et al.  2014(Smolders & de Kort, 2014) | | 28  (16f) | 23±4.1 | Simulated Office  3.2 m $\times$1.8 m | Within | 1000 lx | 8.70 × 10^14 photons/s cm^2^  330 μW/cm^2^ | 1000 | 4000 K | 86 | Polychromatic white  FL | Morning  (9:00, 10:20,11:45) | 92 lx  1.61$\times$10^14^  photons/cm^2^/s  29 μW/cm^2^  4000 K  7 min | 30 m | ✓ | aPVT | ✗ | Auditory Go-no-go | | ↧ | ✗ |
|  |  |  |  |  |  | 200 lx | 1.80 × 10^14 photons/s cm^2^  68 μW/cm^2^ | 200 | 4000 K | 86 |  |  |  |  |  |  |  | Visual 2 back | | ✗ | ↧ |
|  |  |  |  |  |  |  |  |  |  |  |  | Afternoon  (1:15, 2:45, 4:15) |  | 30 m | ✓ | aPVT | ✗ | Auditory Go-no-go | | ↧ | ✗ |
|  |  |  |  |  |  |  |  |  |  |  |  |  |  |  |  |  |  | Visual 2 back | | ✗ | ↧ |
| Vandewalle et al.  2013(Vandewalle et al., 2013) | | 3  (all blind) | 60-67 | Lab | Within | High intensity light | 9.7 × 10^14 photons/s cm^2^  414 μW/cm^2^ | - | - | - | LED | Afternoon  (6 hours after waking up) |  | 2 s | - | EEG | ✓ | - | | - | - |
|  |  |  |  |  |  |  |  |  |  |  |  |  |  |  |  | aPVT | ✓ |  |  |  |  |
| Kaida et al.  2013(Kaida et al., 2013) | | 16  (8f) | 30.7±7.18 | Lab | Within | Bright Light | - | >2000 | 7000-7500 K | 93 | Polychromatic white  FL | Afternoon  14:15-15:30 | <5 lx  1.15 hour | 75 m | ✓ | - | - | Task Switch | | ✗ | ✗ |
|  |  |  |  |  |  | Control | - | <5 | - | - |  |  |  |  |  |  |  |  |  |  |  |
| Kaida et al.  2012(Kaida, Takeda, & Tsuzuki, 2012) | | 15  (7f) | 31.3±7.19 | Lab | Within | Bright Light | - | >2000 | 7000-7500 K | 93 | Polychromatic white  FL | Afternoon  14:15-15:30 | <5 lx  1.15 hour | 75 m | ✓ | - | - | Contextual Cue | | ✗ | ✗ |
|  |  |  |  |  |  | Control | - | <5 | - | - |  |  |  |  |  |  |  | Implicit Learning | | ✗ | ✗ |
| Smolders et al.  2012(Smolders et al., 2012) | | 32  (13f) | 22±4 | Simulated Office  3.6 m $\times$3.2 m | Mixed | Exp2 | - | 1000 | 4000 K |  | Polychromatic white  FL | Morning  (9 am or 11am) | 200 lx (at desk)  4000 K  30 min | 60 m | ✓ | aPVT | ✓ | Letter digit substitute | |  | ✗ |
|  |  |  |  |  |  | Exp1 | - | 200 | 4000 K |  |  |  |  |  |  |  |  |  |  |  |  |
|  |  |  |  |  |  |  |  |  |  |  |  | Afternoon  (1 Pm or ?PM) |  | 60 m | ✓ | aPVT | ✗ | Letter digit substitute | |  | ✗ |
| Lek et al. 2008(Riemersma-Van Der Lek, 2008) | | 180  (170f) | 85.8±5.5 | Group care facilities | Mixed | Bright light |  | ±1000 |  |  | Polychromatic white  FL | Morning  (9am-6pm) | - | - | - | - | - | Cognitive performance | |  | ✓ |
|  |  |  |  |  |  | Dim light |  | ±300 |  |  |  |  |  |  |  |  |  |  |  |  |  |
| Ruger et al.  2006(Rüger et al., 2006) | | 24  (0f) | 23.1±1.5 | Lab | Within | Bright Light | - | 5000 lx | - | - | Bright light boxes | Afternoon  (12 PM- 4PM) | <10 lx | 240 m | ✓ | - | - | - | | - | - |
|  |  |  |  |  |  | Dim Light | - | <10 lx | - | - |  |  |  |  |  |  |  |  |  |  |  |
| Phipps-Nelson et al.  2003(Phipps-Nelson et al., 2003) | | 16  (10f) | 25.3 | Lab | Mixed | Bright light | - | 5000 | - | - | Polychromatic white  FL | Afternoon  (12:00-5:00) | <5 lx | 5 h | ✓ | aPVT | ✓ | - | | - | - |
|  |  |  |  |  |  | Dim light | - | <5 | - | - |  |  |  |  |  | SEM | ✓ |  |  |  |  |
| Akerstedt et al. 2003(Akerstedt, Landstrom, Byström, Nordström, & Wibom, 2003)^2^ | | 20(10f) | F:24.5±4  M: 25.5±4 | Lab | Within | Bright light | - | 2000 | - | - |  | Morning  08:00 | - | 30m | ✓ | EEG | ✗ | - | | - | - |
|  |  |  |  |  |  | Red light | - | 30 | - | - |  |  |  |  |  |  |  |  |  |  |  |
|  |  |  |  |  |  | Dim light | - | 5 | - | - |  |  |  |  |  |  |  |  |  |  |  |
| Daurat  et al. 1993(Daurat et al., 1993) | | 8  (0f) | 22.25±1.26 | Lab | Mixed | Bright light | - | 2000 | - | - |  | Morning  (09:00AM | <150 lx | 24 h | ✗ | EEG | ✗ | Letter  cancellation | | ✗ | ✗ |
|  |  |  |  |  |  | Dim light | - | 150 | - | - |  |  |  |  |  |  |  |  |  |  |  |
| Badia et al. 1991(Badia, Myers, Boecker, Culpepper, & Harsh, 1991) | | 44  (0f) | 18-32 | Lab | Between  group | Bright light | - | (5000 – 10000 ) lx^2^ | - | - |  | Afternoon  13.00 | - | 90 m | ✗ | EEG | ✗ | Digit recall | | - | ✗ |
|  |  |  |  |  |  |  |  |  |  |  |  |  |  |  |  |  |  | Two-letter search | | - | ✗ |
|  |  |  |  |  |  | Dim light | - | 50 lx | - | - |  |  |  |  |  |  |  | Two-column addition | | - | ✗ |
|  |  |  |  |  |  |  |  |  |  |  |  |  |  |  |  |  |  | Serial addition subtraction | | - | ✗ |
|  |  |  |  |  |  |  |  |  |  |  |  |  |  |  |  |  |  | Continuous Performance task | | - | ✗ |

*Note:* ^1^ Exposure duration per session/ condition

^2^ Whether Illuminance is reported at desk level or eye level is unclear
