# Supplementary material for: Effects of Daytime Electric Light Exposure on Human Alertness and Higher Cognitive Functions: A Systematic Review
Source: Front Psychol. 2022 Jan 5;12:765750. doi: 10.3389/fpsyg.2021.765750 (PMC8766646; doi:10.3389/fpsyg.2021.765750)
Supplement: Supplementary file 4 [file Table_3.docx]

**Supplementary Table 3**

*Effects of daytime electric light with* ***high MDER*** *light exposure on alertness and higher cognitive functions. The "✓" indicates that a significant beneficial influence is reported, “↧” indicates that a deteriorate influence is reported and "✗"indicates that no significant effect is reported.*

| **Author (year)** | **Participants** | **Age** | **Focus** | **Settings** | **Design** | **Light Manipulation** | **Radiometric Properties** | **Colorimetric Properties** | **Photopic lux** | **MEDI** | **MDER** | **Temporal patter** | | | **Alertness** | | | **Higher cognitive functions** | | |
| --- | --- | --- | --- | --- | --- | --- | --- | --- | --- | --- | --- | --- | --- | --- | --- | --- | --- | --- | --- | --- |
|  |  |  |  |  |  |  |  |  |  |  |  | **Time** | **Baseline^2^** | **Duration^1^** | **Sub. Alertness** | **Objective alertness** | | **Task** | **Performance** | |
|  |  |  |  |  |  |  |  |  |  |  |  |  |  |  |  | **Task** |  |  | **RT** | **ACC** |
| Grant et al. (2021)(Grant et al., 2021) | 39 (21f) | 24.5±3.2 | Spectrum (Polychromatic LED) | Lab | Between | Daylight like spectra high melEDI | 19.03 μW/cm^2^ | 5251 K | 50 | 45.36 | 0.91 | Morning (2 hour after awakening) | ~3 lx | 8 h | ✓ | PVT | ✗ | Addition task | - | ✓ |
|  |  |  |  |  |  | Conventional spectra high | 18.06 μW/cm^2^ | 4864 K | 50 | 38.21 | 0.76 |  |  |  |  |  |  | MST | - | ✓ |
|  |  |  |  |  |  | melEDI  Daylight like spectra low melEDI | 17.35 μW/cm^2^ | 3127 K | 50 | 27.17 | 0.54 |  |  |  |  |  |  |  |  |  |
|  |  |  |  |  |  | Conventional Low melEDI | 15.11 μW/cm^2^ | 2984 K | 50 | 24.02 | 0.48 |  |  |  |  |  |  |  |  |  |
| Choi et al. 2020(Choi & Suk, 2020) | 23 (11f) | 18.61±0.50 | Spectrum (LED) | Lab | Within | Blue enriched white light | **-** | 6575K;  Ra=96 | 1000 | 1033.51 | 1.03 | Day time | 1 m | 10 m | - | EEG  Alpha wave | ✗ | N-back | ✗ | ✗ |
|  |  |  |  |  |  | Neutral | **-** | 5127K;  ra = 98 | 1000 | 863.23 | .86 |  |  |  |  |  |  |  |  |  |
|  |  |  |  |  |  |  |  |  |  |  |  |  |  |  |  | EEG  beta wave | ✓ |  |  |  |
|  |  |  |  |  |  | Light's off scenario | **-** | **-** | <1 | **-** |  |  |  |  |  | ECG | ✓ |  |  |  |
| Cajochen et al. 2019(Cajochen et al., 2019) | 15 (0f) | 23.2±4.3 | Spectrum (polychromatic LED) | Lab | Within | dayLED | - | 4000 K;  ra= 99 | 100 | 76.17 | 0.76 | Morning (8 -24) | 100 lx | 16h | ✓ | aPVT | ✗ | N-back |  | ✗ |
|  |  |  |  |  |  | conLED | - | 4000 K;  ra =79 | 100 | 62.59 | 0.62 |  |  |  |  |  |  |  |  |  |
| Ru et al. 2019(Ru et al., 2019) | 57 (38f) | 20.23±1.58 | Interaction of Intensity and CCT  (Polychromatic LED) | Simulated office  3.6 by 3.6 m | Mixed | 6500 K 1000 lx | 331 μW/cm^2^  9.28× 10^14^ photons/s cm^2^ | 6500 K;  ra=81 | 1000 | 903.98 | .90 | Morning (10:00-17:00) | 70-73 lx | 50 m | ✗^4^ | PVT | ✗^4^ | Go-no-go | ✗^4^ | ✗^4^ |
|  |  |  |  |  |  |  |  |  |  |  |  |  |  |  |  |  |  | Flanker test | ✗^4^ | ✗^4^ |
|  |  |  |  |  |  | 6500 K 100 lx | 33 μW/cm^2^;  8.99× 10^13^ photons/s cm^2^ | 6500 K;  ra=81 | 100 | 86.69 | .86 |  |  |  |  |  |  |  |  |  |
|  |  |  |  |  |  |  |  |  |  |  |  |  |  |  |  |  |  | PVSAT | ✗^4^ | ✗^4^ |
|  |  |  |  |  |  | 3000 K 1000 lx | 324 μW/cm^2^;  9.54 × 10^14^ photons/s cm^2^ | 3000 K;  ra=83 | 1000 | 483.70 | 0.48 |  |  |  |  |  |  |  |  |  |
|  |  |  |  |  |  | 3000 K 100 lx | 31 μW/cm^2^;  9.07 × 10^13^ photons/s cm^2^ | 3000 K;  ra=83 | 100 | 50.72 | 0.51 |  |  |  |  |  |  |  |  |  |
| -Zeeuw el al. 2019(Zeeuw et al., 2019) | 82 (45f) | 24.4±2.7 | Interaction of Intensity and CCT  (Polychromatic LED) | Lab | Mixed | Low illuminance Highest mel | 47.0 μW/cm^2^  1.3× 10^14^ photons/s cm^2^ | 480nm | 103.6 | 149.45 | 1.4 | Moring 11.50 am | <5 lx  50 min | 180 | ✓ | EEG | ✓ | - | - | - |
|  |  |  |  |  |  | 200 lx High mel | 88.4 μW/cm^2^  2.6 × 10^14^ photons/s cm^2^ | 480nm | 210.5 | 190.67 | 0.98 |  |  |  |  |  |  |  |  |  |
|  |  |  |  |  |  | 600 lx High mel | 266.2 μW/cm^2^  7.8× 10^14^ photons/s cm^2^ | 480nm | 628.5 | 569.29 | 0.96 |  |  |  |  |  |  |  |  |  |
|  |  |  |  |  |  | 12000 lx High mel | 528.6 μW/cm^2^  1.6× 10^15^ photons/s cm^2^ | 480nm | 1259 | 1140.40 | 0.96 |  |  |  |  |  |  |  |  |  |
|  |  |  |  |  |  | Low illuminance high mel | 41.2 μW/cm^2^  1.2× 10^14^ photons/s cm^2^ | 480nm | 103.2 | 84.42 | 0.82 |  |  |  |  |  |  |  |  |  |
|  |  |  |  |  |  | 200 lx low mel | 63.6 μW/cm^2^  1.8× 10^14^ photons/s cm^2^ | 435nm | 110.3 | 99.90 | 0.50 |  |  |  |  |  |  |  |  |  |
|  |  |  |  |  |  | 600 lx low mel | 187.5 μW/cm^2^  5.4× 10^14^ photons/s cm^2^ | 435nm | 329.3 | 298.27 | 0.50 |  |  |  |  |  |  |  |  |  |
|  |  |  |  |  |  | 12000 lx low mel | 376.4 μW/cm^2^  1.1× 10^14^ photons/s cm^2^ | 435nm | 659 | 596.92 | 0.50 |  |  |  |  |  |  |  |  |  |
|  |  |  |  |  |  | Low illuminance low mel | 33.1 μW/cm^2^  9.6× 10^13^ photons/s cm^2^ | 435nm | 99.7 | 49.55 | 0.50 |  |  |  |  |  |  |  |  |  |
|  |  |  |  |  |  | Dim light |  |  | <5 |  |  |  |  |  |  |  |  |  |  |  |
| Askaripoor et al. 2019(Askaripoor et al., 2019) | 20 (0f) | 27.65±3.65 | Spectrum (polychromatic dimmable fluorescent light) | simulated office environment area 19m^2^ | Within | Blue enriched white light | 121 μW/cm^2^;  3.18 × 10^14^ photons/s cm^2^ | 12000 K;  ra= 83 | **317** | **351.45** | **1.1** | Afternoon 13:50 | 13 m  <5 lx  3520 K | 130 m | ✓^3^ | EEG | ✓ | CPT | ✓^3^ | ✗ |
|  |  |  |  |  |  |  |  |  |  |  |  |  |  |  |  |  |  | Go-no-go | ✓^3^ | - |
|  |  |  |  |  |  | Red saturated white light | 96 μW/cm^2^;  2.7 × 10^14^ photons/s cm^2^ | 4000 K;  ra=82.9 | 332 | 176.63 | 0.53 |  |  |  |  |  |  | 2 back | ✓^3^ |  |
|  |  |  |  |  |  |  |  |  |  |  |  |  |  |  |  |  |  |  |  |  |
|  |  |  |  |  |  | Normal White light | 92 μW/cm^2^;  2.7 × 10^14^ photons/s cm^2^ | 2700 K;  ra = 81.9 | 333 | 104.17 | 0.31 |  |  |  |  |  |  |  |  |  |
|  |  |  |  |  |  | Dim light |  |  | <5 |  |  |  |  |  |  |  |  |  |  |  |
| Askaripoor et al. 2018(Askaripoor et al., 2018) | 22 (0f) | 27.32±3.63 | Spectrum (polychromatic dimmable fluorescent) | Lab 3.4 $\times$5.6$\times$3.05m | Within | 7343 K | 117 μW/cm^2^;  3.14 × 10^14^ photons/s cm^2^ | 7340 K;  ra=86.9 | 333 | 309.78 | .93 | Morning 8:30 | 13m  5 lx, 3520K | 80m | ✓^3^ | EEG | ✓^3^ | CPT | ✓^3^ | ✗ |
|  |  |  |  |  |  |  |  |  |  |  |  |  |  |  |  | HR | ✓^3^ |  |  |  |
|  |  |  |  |  |  | 3730 K | 96 μW/cm^2^;  2.7 × 10^14^ photons/s cm^2^ | 3730 K;  ra=82.9 | 332 | 176.63 | 0.53 |  |  |  |  |  |  |  |  |  |
|  |  |  |  |  |  |  |  |  |  |  |  | Afternoon 13:50 | 13m  5 lx, 3520K | 80m | ✓^3^ | EEG | ✓^3^ | CPT | ✓^3^ | ✗ |
|  |  |  |  |  |  | 2564 k | 92 μW/cm^2^;  2.7 × 10^14^ photons/s cm^2^ | 2564 K;  ra=81.9 | 333 | 104.16 | 0.31 |  |  |  |  | HR | ✓^3^ |  |  |  |
|  |  |  |  |  |  | Dim light | <5 lx |  | <5 lx |  | 0 |  |  |  |  |  |  |  |  |  |
| Rodriguez-Morilla et al. 2018(Rodriguez-Morilla et al., 2018) | 17 (11f) | 20.5±1.48 | Spectrum  (Polychromatic LED) | Lab | Within | Blue enriched white light | 141.14 μW/cm^2^;  4 × 10^14^photons/s cm^2^ | 490 nm | 469 | 203.66 | 0.43 | Morning  8:30-9:30 | 30 m  Dark adaptation | 1 h | - | PVT | ✓ | Simulated  Driving  task | ✓ |  |
|  |  |  |  |  |  | Dim light | No light |  |  |  |  |  |  |  |  |  |  |  |  |  |
| Lok et al. 2018(Lok, Woelders, et al., 2018) | 50 (25f) | 23.02±0.29 | Intensity  (Polychromatic fluorescent) | Lab | Mixed | 222 lx | - | - | 222 | 198.37 | 0.89 | Morning  7:30 | <10 lx | 60 m | - | Blink  parameters | ↧ | Go-no go | ✗ | ↧ |
|  |  |  |  |  |  | 666 lx | - | - | 666 | 581.52 | 0.87 |  |  |  |  |  |  |  |  |  |
|  |  |  |  |  |  | 74 lx | - | - | 74 | 60.69 | 0.82 |  |  |  |  |  |  |  |  |  |
|  |  |  |  |  |  | 24 lx | - | - | 24 | 19.02 | 0.89 |  |  |  |  |  |  |  |  |  |
| Hartstein et al 2017(Hartstein, Durniak, Karlicek, & Berthier, 2017) | 40 (21f) | 20.5  (18-26) | Spectrum  (Polychromatic fluorescent & LED) | Lab 2.6$\times$2.2m | Mixed | Cool Light |  | 5000 K | 350 | 500.72 | 1.4 | Morning  9:00 or 10:00 | 20 min |  | - | - | - | Mental  Rotation test | ✗ | ✗ |
|  |  |  |  |  |  |  |  |  |  |  |  |  |  |  |  |  |  | Go-no-go test | ✗ | ✗ |
|  |  |  |  |  |  | Warm light |  | 3500 K | 350 | 319.59 | 0.9 |  |  |  |  |  |  | Task-switching | ✗ | ✗ |
| Smolders et al 2017(Smolders & de Kort, 2017) | 39 (12f) | 23±3.9 | Spectrum (Polychromatic fluorescen) | Simulated office 3.93$\times$7m | Mixed | 6000 k | 41 μW/cm^2^;  .11 × 10^14^ photons/s cm^2^ | 6000 K | 124 | 100.54 | 0.81 | Morning  9:00 or 11:00 | 4000K  158lx | 60 m | ✗ | aPVT | ✗ | Addition  task | - | ✗ |
|  |  |  |  |  |  |  |  |  |  |  |  |  |  |  |  | HR | ↧ | Letter cancellation task | ✗ | ↧ |
|  |  |  |  |  |  |  |  |  |  |  |  |  |  |  |  | Skin conductance | ✗ | ANT  (Executive) | ✗ | ✗ |
|  |  |  |  |  |  |  |  |  |  |  |  |  |  |  |  | ANT  (attention | ✗ |  |  |  |
|  |  |  |  |  |  |  |  |  |  |  |  | Afternoon  13:00 or 15:00 | 4000K  158lx | 60 m | ✗ | aPVT | ✗ | Addition  task | - | ↧ |
|  |  |  |  |  |  |  |  |  |  |  |  |  |  |  |  | HR | ✗ | Letter cancellation task | ✗ | ✗ |
|  |  |  |  |  |  | 2700 k | 39 μW/cm^2^;  1.11 × 10^14^ photons/s cm^2^ | 2700 K | 132 | 62.5 | 0.47 |  |  |  |  |  |  |  |  |  |
|  |  |  |  |  |  |  |  |  |  |  |  |  |  |  |  | Skin conductance | ✗ |  |  |  |
|  |  |  |  |  |  |  |  |  |  |  |  |  |  |  |  |  |  | ANT  (Executive) | ✗ | ✗ |
|  |  |  |  |  |  |  |  |  |  |  |  |  |  |  |  | ANT  (attention | ✗ |  |  |  |
| Te Kulve  et al. 2017(te Kulve et al., 2017) | 19 (0f) | 22.3±1.9 | Intensity  (Polychromatic LED) | Lab | Within | Bright Light | - | 4000 K | 986 | 690.31 | 0.70 | Morning  8:00 | 30 m  250 lx  4000 K | 75m | ✓ | PVT | ✗ | - | - | - |
|  |  |  |  |  |  | Dim light | - | 4000 K | 4.13 | 2.63 | 0.60 |  |  |  |  |  |  |  |  |  |
| Huiberts et al. 2016(Huiberts et al., 2016) | 39 (28f) | 21.2±2.1 | Intensity (Polychromatic fluorescent) | Simulated office 3.9m$\times$7.4m | Mixed | 165 lx | 51 μW/cm^2^;  1.34 × 10^14^  photons/s cm^2^ | 4700 K;  ra=87 | 165 | 117.75 | 0.71 | Morning  9:00-10:30 | 25m  120 lx | 90 m | - | aPVT | ✗ | BDST | - | ✗ |
|  |  |  |  |  |  |  |  |  |  |  |  |  |  |  |  | HR | ✗ |  |  |  |
|  |  |  |  |  |  | 600 lx | 188 μW/cm^2^;  4.96 × 10^14^  photons/s cm^2^ | 4700 K;  ra=87 | 600 | 421.2 | 0..70 |  |  |  |  |  |  |  |  |  |
|  |  |  |  |  |  |  |  |  |  |  |  | Afternoon  15:45-17:15 | 25m  120 lx | 90 m | - | aPVT | ✗ | BDST | - | ✗ |
|  |  |  |  |  |  | 1700 lx | 530 μW/cm^2^;  1.39 × 10^15^  photons/s cm^2^ | 4700 K;  ra=87 | 1700 | 1147.65 | 0.67 |  |  |  |  | HR | ✗ |  |  |  |
| Huiberts et al. 2015(Huiberts et al., 2015) | 64 (32f) | 21.4±2.1 | Intensity (Polychromatic fluorescent) | Lab | Mixed | 4000 K 200 lx | 61 μW/cm^2^;  1.63 × 10^14^  photons/s cm^2^ | 4000 K;  ra=87 | 200 | 130.43 | 0.65 | Morning  (9:00-10:30;10:45-12:15) | 100 lx | 60 m | ↧ | - | - | Forward  Digit Span | - | ↧ |
|  |  |  |  |  |  |  |  |  |  |  |  |  |  |  |  |  |  | Backward digit Span | - | ↧ |
|  |  |  |  |  |  |  |  |  |  |  |  |  |  |  |  |  |  | 1-Back | ✗ | ✗ |
|  |  |  |  |  |  |  |  |  |  |  |  |  |  |  |  |  |  | 2-back | ✗ | ✗ |
|  |  |  |  |  |  | 4000 K1000 lx | 304 μW/cm^2^;  8.09 × 10^14^  photons/s cm^2^ | 4000 K;  ra=87 | 1000 | 621.38 | 0.62 |  |  |  |  |  |  | 3-back | ✗ | ✗ |
|  |  |  |  |  |  |  |  |  |  |  |  | Afternoon  (12:15-13:45; 13:45-15:15) | 100 lx | 60 m | ↧ | - | - | Forward  Digit Span | - | ↧ |
|  |  |  |  |  |  |  |  |  |  |  |  |  |  |  |  |  |  | Backward digit Span | - | ↧ |
|  |  |  |  |  |  |  |  |  |  |  |  |  |  |  |  |  |  | 1-Back | ✗ | ✗ |
|  |  |  |  |  |  |  |  |  |  |  |  |  |  |  |  |  |  | 2-back | ✗ | ↧ |
|  |  |  |  |  |  |  |  |  |  |  |  |  |  |  |  |  |  | Forward  Digit Span | ✗ | ✗ |

*Note:* ^1^ Exposure duration per session/ condition

^2^Reported at eye level

^3^Compared to dim light

^4^Interaction effect of illuminance & spectral composition.
